# Standardized Effect Measures Informing Next‐Generation Strategies for Mechanical Stimulation in Cartilage Tissue Engineering

**DOI:** 10.1002/adhm.71309

**Published:** 2026-06-03

**Authors:** Jiaqi K. Shen, Tony B. Huang, Catherine E. Davey, Elias Salzer, Gerjo J. V. M. Van Osch, Sandra J. Shefelbine, Marcus G. Pandy, Kathryn S. Stok

**Affiliations:** ^1^ Department of Biomedical Engineering The University of Melbourne Parkville Victoria Australia; ^2^ Department of Orthopaedics and Sports Medicine Erasmus MC University Medical Center Rotterdam Rotterdam The Netherlands; ^3^ Department of Otorhinolaryngology Head and Neck Surgery Erasmus MC University Medical Center Rotterdam Rotterdam The Netherlands; ^4^ Department of Biomedical Engineering University of Technology Delft The Netherlands; ^5^ Department of Mechanical and Industrial Engineering Northeastern University Boston Massachusetts USA; ^6^ Department of Bioengineering Northeastern University Boston Massachusetts USA; ^7^ Department of Mechanical Engineering The University of Melbourne Parkville Victoria Australia

**Keywords:** bioreactor, cartilage mechanobiology, cartilage tissue engineering, mechanical stimulation, mechanoadaptation

## Abstract

Dynamic mechanical stimulation provides cues essential to mechanoadaptation, influencing extracellular matrix composition and functional properties. Cartilage tissue engineering implements a wide spectrum of stimulation modalities and loading parameters, yet the absence of standardization hinders direct comparison and limits investigation of optimal mechanical stimulation protocols. This systematic review summarizes published parameters of mechanical stimulation and applies standardized effect measures to compare their efficacy on matrix production in tissue‐engineered cartilage. A total of 95 in vitro studies were included, covering six stimulation modalities (compression, tension, shear, hydrostatic pressure, fluid‐induced shear, and combined stimuli) and chondrogenic outcomes (aggrecan and collagen II gene expression, glycosaminoglycan and collagen deposition, and compressive equilibrium modulus). The combined application of compression and shear was most effective, suggesting that complex loading patterns are potentially more beneficial for optimal cartilage mechanoadaptation. Loading dynamics and magnitude correlated with chondrogenic outcomes in meta‐regression analysis, particularly for fluid‐induced shear, which exhibited decreasing effects at higher intensities. Standardized effect measures enabled cross‐study comparison despite wide methodological variability. A comprehensive in vitro comparison under rigorously controlled culture conditions, with precise understanding of sub‐tissue mechanical stimuli, is essential for improving research reproducibility, optimizing mechanical microenvironments, and guiding bioreactor design for enhanced cartilage matrix development.

## Graphical abstract

This systematic review quantitatively compares conventional mechanical stimulation strategies in cartilage tissue engineering across 85 heterogeneous in vitro studies. Applying standardized effect measures, meta‐analysis reveals that combined compression and shear loading optimally promotes cartilage matrix development. These findings establish a quantitative baseline for mechanically stimulated chondrogenesis, guiding future stimulation protocol optimization and next‐generation bioreactor design.

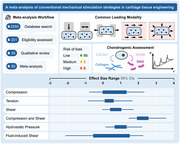

## 1 Introduction

Cartilage is a mechanosensitive tissue capable of modifying its extracellular matrix in response to dynamic changes in the mechanical microenvironment. Mechanical loading propagates into the extracellular space, where mechano‐transducers on chondrocytes, such as ion channels, integrin receptors and primary cilia, are activated and convert dynamic mechanical cues into biochemical signals that regulate chondrogenesis through mechanotransduction pathways [1, 2, 3]. For instance, mechanical activation of the TRPV4 ion channel has been shown to influence cartilage matrix development through intracellular signaling cascades such as calcium flux [4, 5].

**FIGURE 1:**
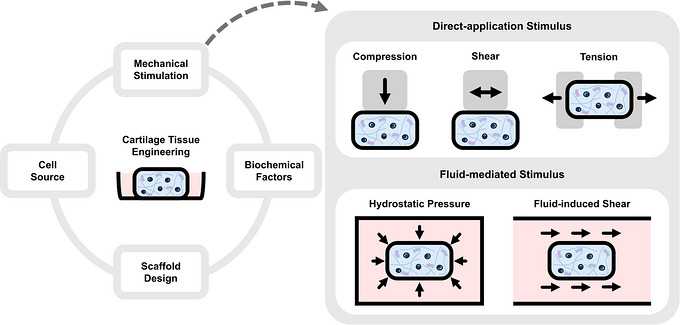
(Left) Overview of four fundamental pillars that support cartilage tissue engineering, and (Right) dynamic mechanical stimulation regimes conventionally applied to induce cartilage mechanoadaptation. Each stimulus regime or a combination of stimuli induces unique dynamic changes in the mechanobiological environment that facilitates cartilage matrix development, ultimately leading to the formation of functional cartilage tissue.

Beyond the three traditional pillars that support cartilage tissue engineering – cells, scaffolds, biochemical factors – mechanical stimulation has emerged as another critical determinant for successful tissue outcomes [6, 7]. Although detailed mechanisms of cartilage mechanoadaptation still require further characterization [8], there is convincing evidence that conventional mechanical stimuli generally promote cartilage biosynthesis, including direct application of compression, shear, and tension, as well as fluid‐mediated pressure such as hydrostatic pressure and fluid‐induced shear (Figure 1) [9, 10].

Inspired by physiological loading conditions of articular cartilage, dynamic mechanical stimulation such as cyclic compression has been shown to upregulate matrix metabolism compared to static culture, the latter typically suppressing cartilage biosynthesis in tissue constructs [11, 12]. Growing interest in this area has led to the development of specialized tissue cultivation systems, known as bioreactors. Custom‐built bioreactors provide controlled mechanical stimulus while supplying adequate in vitro culture conditions, tailored to various research objectives [13]. However, extensively diversified cartilage research and user‐defined mechanical stimulation regimes create significant variability in tissue outcomes, hampering researcher consensus on mechanical stimulation parameters and assessment methods for reproducible chondrogenic results [14]. The lack of standardization in cartilage tissue engineering presents challenges for quality control and research translation [15, 16].

The application of mechanical stimulus in bioreactors for cartilage tissue engineering has been qualitatively reviewed in the past [17, 18, 19, 20], however, an evidence‐based approach is required for quantitatively characterizing the efficacy and heterogeneity amongst the bioreactor studies. Systematic literature reviews have gained increasing popularity as a means to comprehensively synthesize non‐standardized findings to promote reproducibility [21]. Previously, Bleuel et al. (2015) qualitatively summarized chondrogenic effects of cyclic tension in tissue‐engineering studies, and Hodder et al. (2020) incorporated meta‐analysis to quantitatively compare chondrogenic effects of hydrostatic pressure at different control parameters, both in systematic reviews [22, 23]. However, comparison across various mechanical stimulation regimes is also needed to gain a more comprehensive understanding of conventional practices used in cartilage tissue engineering research.

This systematic review aims to address the following questions: (1) What control parameters of dynamic mechanical stimulation are applied to enhance chondrogenesis in tissue‐engineered articular cartilage? and (2) Can trends or patterns be identified from the published studies about chondrogenic effects of mechanical stimulation? A synthesis of mechanical stimulation parameters from published studies of cartilage tissue engineering is provided, and a meta‐analysis is then performed to quantitatively compare chondrogenic effect sizes across conventional stimulation modalities. This critical evaluation of the cartilage tissue engineering field from the perspective of mechanobiology will help understand the essential mechanical signals required for developing a successful tissue‐engineered cartilage and highlight important guidance required for standardization in heterogeneous in vitro studies.

## 2 Material and Methods

### 2.1 Protocol and Registration

This systematic review followed a pre‐defined protocol in accordance with the PRISMA 2020 statement [24]. The systematic review protocol was registered on the Open Science Framework registries and is publicly available at https://doi.org/10.17605/OSF.IO/NHEUF [25].

### 2.2 Eligibility Criteria

Literature screening was conducted based on pre‐established inclusion and exclusion criteria. A study was eligible for inclusion if: (1) experiments were conducted in vitro; (2) target tissue type was hyaline articular cartilage; (3) dynamic mechanical stimulation was used for tissue culture; (4) quantitative assessment of matrix production was conducted specific to hyaline articular cartilage; (5) the paper is a journal article of primary research; and (6) the paper is written in English. A study was excluded if: (1) cartilage explant was the only tissue sample used; (2) in‐study control (unloaded) groups were not present; or (3) no experimental data were available. Cell source, scaffold design and biochemical factors were not specified as eligibility criteria, as this review is focused on studies that investigated the role of mechanical stimulation as a critical determinant in cartilage tissue engineering.

### 2.3 Information Sources

Scopus, MEDLINE, Ei Compendex and EMBASE databases were selected for literature search.

### 2.4 Search Strategy

Details of the search strategy can be found in Tables S1–S4. The latest search was conducted on August 25, 2025.

### 2.5 Study Selection

The term “study” in this review refers to the individual journal article and research paper. Study selection was conducted in the following phases: (1) database search with the developed search strategy; (2) removal of duplicate studies; (3) initial screening by title and abstract; and (4) full‐text screening based on the eligibility criteria. Studies were independently screened by two reviewers (JKS, TBH), and the results were compared to resolve disagreements. Any unresolved disagreements were decided by a third reviewer (KSS). The study selection process was managed using an online systematic review platform, Covidence (Veritas Health Innovation, Melbourne, Australia), which automatically removed most duplicate studies with minor manual removal. The inter‐rater reliability during study screening was evaluated by percentage agreement and Cohen's kappa [26].

### 2.6 Data Extraction

Data extraction was independently conducted by a primary reviewer (JKS) in Covidence. All extracted quantitative data were confirmed by a secondary reviewer (TBH) and exported to Excel for further data analysis. In studies where outcome data were only available in figures, data were digitally extracted using graph digitizing software PlotDigitizer (https://plotdigitizer.com). Collected data items are listed in Table S5.

### 2.7 Risk of Bias Assessment

The risk of bias assessment was conducted for quality control, using the Quality Assessment Tool for In Vitro Studies (QUIN Tool) by Sheth et al. (2024) [27]. Assessment criteria from the QUIN Tool were customized to capture key risks relevant to this review, while non‐applicable criteria were omitted. Studies were rated according to 8 criteria: clarity of stated objectives, specification of sample size, details of study population, details of comparison group, detailed explanation of study design, specification of standardization methods for outcome measurement, details of statistical analysis, and presentation of results. Two reviewers (JKS, TBH) independently scored each criterion from 2 to 0 (2, adequately satisfied; 1, inadequately satisfied; 0, not satisfied) and calculated a final score in percentage to rate the risk of bias in terms of high (<50%), medium (50%–70%) or low (>70%). Reviewer disagreements were first discussed, and when consensus could not be reached, a third reviewer (KSS) provided the final decision.

### 2.8 Data Synthesis

#### 2.8.1 Effect Size

Effect size was calculated using standardized mean difference (SMD) to calculate the generalized effect estimation of mechanical stimulation, assuming variables such as biochemical stimuli, cell source, scaffold design, and culture environment introduce random variance. The pooled standard deviation of chondrogenic outcome data (aggrecan gene expression, glycosaminoglycan deposition, collagen II gene expression, collagen deposition, and compressive equilibrium modulus) was first computed with Equation (1) from the unloaded group (control) and the mechanically stimulated group (treatment). SMD was first calculated by applying Cohen's *d*, using Equation (2), and then corrected by computing Hedges’ *g* with Equation (3) to account for small sample bias:

(1) $$s_{\mathrm{pooled}}=\sqrt{\frac{\left(n_{1}-1\right)\cdot s_{1}^{2}+\left(n_{2}-1\right)\cdot s_{2}^{2}}{n_{1}+n_{2}-2}}\;$$

(2) $$d=\frac{\bar{x_{1}}-\bar{x_{2}}}{s_{\mathrm{pooled}}}\;$$

(3) $$g=d\times \left(1-\frac{3}{4\left(n_{1}+n_{2}\right)-9}\right)$$

where *n*_1_ and *n*_2_ are sample sizes of the treatment group and the control group, respectively, *s*_1_ and *s*_2_ are the standard deviations of chondrogenic outcome in the treatment group and control group, respectively, *s*_pooled_ is the pooled standard deviation in both groups combined, and $\bar{x_{1}}$ and $\bar{x_{2}}$ are the means of chondrogenic outcomes from the treatment group and control group, respectively.

#### 2.8.2 Statistical Analysis

The number of distinct studies was labelled with *n*. The number of distinct mechanical stimulation groups (excluding control groups) or protocols was labelled with *k* since a single study can contain multiple mechanical stimulation groups.

Subgroup analysis was conducted to estimate the grouped effects of all study groups within each pre‐defined category based on specific study‐level characteristics. A random‐effects model was applied with the Restricted Maximum Likelihood (REML) method to estimate the within‐study variance ($\sigma_{i}^{2}$) and between‐study variance (τ^2^) [28], following the form of a general linear mixed‐effects model with heteroscedastic errors, as shown in Equation (4):

(4) $$y_{i}=\mu +u_{i}+\varepsilon_{i}$$

where *y_i_* is the observed effect size in study *i*, μ is the overall true effect size, $u_{i}∼N\left(0,\tau^{2}\right)$, and $\varepsilon_{i}∼N\left(0,\sigma_{i}^{2}\right)$.

The proportion of statistical heterogeneity within each subgroup was quantified using *I* ^2^ statistics [29]. Studies that did not report standard deviation or standard error of the mean of control data were assumed to share the same variability between the treatment and the control group. Outliers of the calculated SMD were defined as data points with Z‐scores exceeding ±3 standard deviations from the mean and were excluded from the analysis. Publication bias, or small‐study effect, was assessed using Egger's test, which detects funnel plot asymmetry by performing a linear regression of the standardized effect size against the associated precision and testing whether the regression intercept (*b*) significantly differed from zero (*p* < 0.05) [30].

#### 2.8.3 Data Analysis Software

Data analyses, including subgroup analysis, forest plots, funnel plots, and Egger's tests for publication bias, were conducted in RStudio (R version 4.4.2) using in‐house R scripts and packages of metafor, dplyr, and ggplot2 [31, 32, 33].

## 3 Results

### 3.1 Study Selection

**FIGURE 2:**
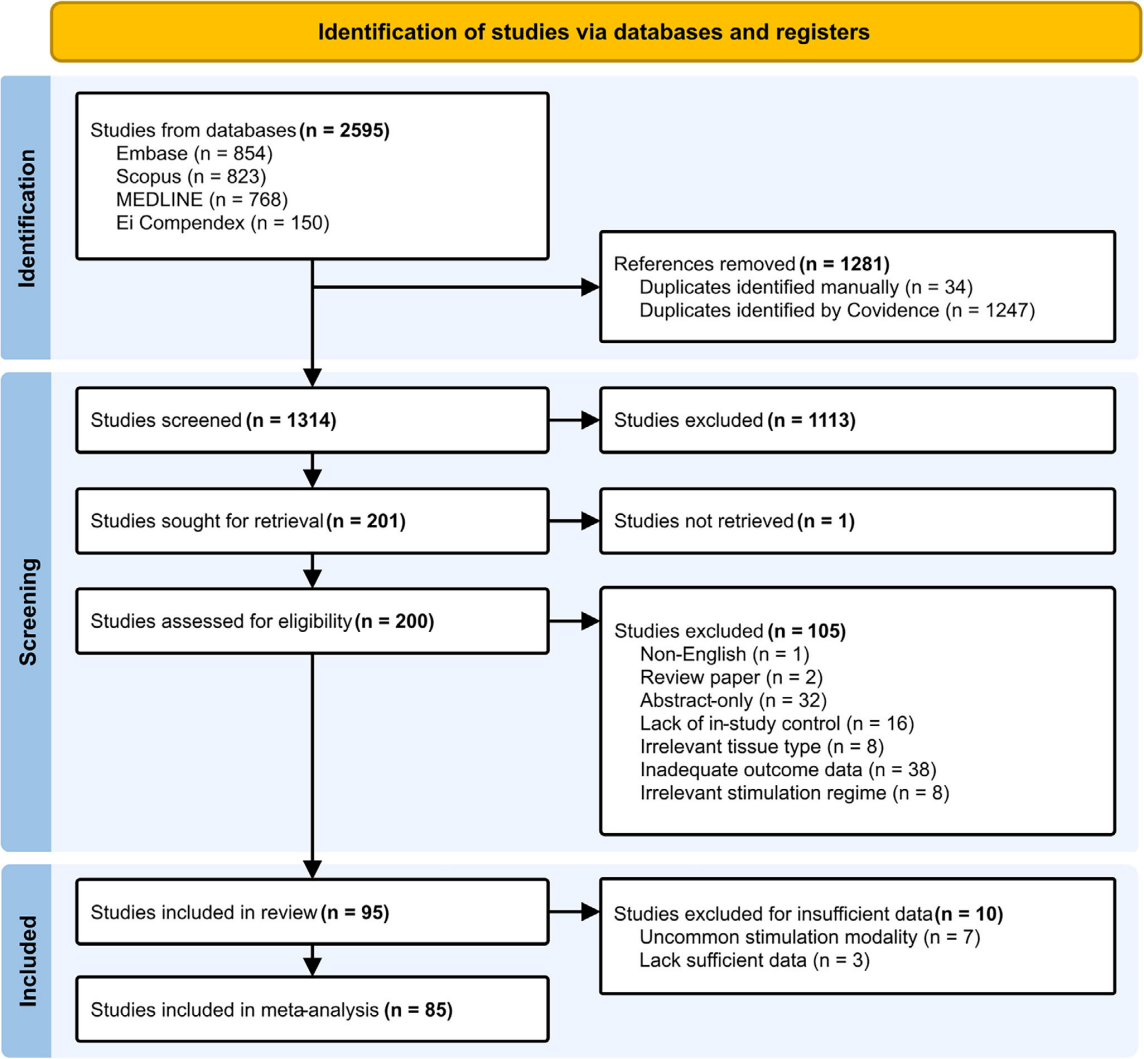
PRISMA flow diagram of study selection.

A total of 1314 studies were identified from the database search. After screening by abstract and title and assessing by eligibility criteria, 95 studies were included for qualitative synthesis, of which 85 studies were included for meta‐analysis (Figure 2) [12, 34, 35, 36, 37, 38, 39, 40, 41, 42, 43, 44, 45, 46, 47, 48, 49, 50, 51, 52, 53, 54, 55, 56, 57, 58, 59, 60, 61, 62, 63, 64, 65, 66, 67, 68, 69, 70, 71, 72, 73, 74, 75, 76, 77, 78, 79, 80, 81, 82, 83, 84, 85, 86, 87, 88, 89, 90, 91, 92, 93, 94, 95, 96, 97, 98, 99, 100, 101, 102, 103, 104, 105, 106, 107, 108, 109, 110, 111, 112, 113, 114, 115, 116, 117, 118, 119, 120, 121, 122, 123, 124, 125, 126, 127]. Inter‐rater reliability was high, with percentage agreement of 88.9% during title‐and‐abstract screening and 85.0% during full‐text screening. Because percentage agreement does not account for chance agreement, Cohen's kappa was also calculated, with values of 0.619 and 0.699 for title‐and‐abstract and full‐text screening, respectively. According to McHugh's scale [128], both Cohen's kappa suggests moderate agreement between the two reviewers (JKS, TBH) during screening.

### 3.2 Risk of Bias Assessment

The risk of bias was low overall, supported by a mean score of 89.47% according to the QUIN scale. Mean scores for each assessment criterion are summarized in Figure S1a. Among all criteria, inadequate specification of sample size contributed most to the overall risk of bias. 79 out of 95 studies used a sample size less or equal to 10, and 30 studies used a sample size of 3, suggesting a potential risk of unstable effect measure with large variance. The distribution of scores across all included studies indicated that none was rated as high risk of bias, thereby fulfilling quality assessment; 4 of the 95 studies were rated as medium risk, and the remainder as low risk (Figure S1b).

### 3.3 Study Characteristics

Details of study characteristics for each included study are summarized in Table S6.

#### 3.3.1 Control Parameters of Mechanical Stimulation

Control parameters specified by the users can vary according to the bioreactor type and specific experimental setups, and thereby result in multiple units across studies (Table 1). For example, the amplitude of direct compression was specified by percentage of strain, mN, mbar, or cm, depending on whether the compressive load was position‐controlled, force‐controlled, pneumatically controlled, or height‐controlled, respectively. Additionally, the unit of fluid‐induced shear was specified in either rotations per minute (rpm) or mL/min, when induced in an orbital shaker or continuous perfusion system, respectively. Only 3 studies reported the loading rate used to reach the corresponding loading amplitude, which were 0.2% strain/second for compression [36], 1 mm/s for direct tension [110], and 60°/s for centrifugal force [113].

**TABLE 1:** Control parameters applied for each mechanical stimulation modality.

Stimulation Modality	Preload^a^	Amplitude^a^, ^c^	Frequency	Flow Rate	Daily Loading Time	Study Duration^b^	Study Count
Direct‐application stimulation							
Compression	5%–50% strain	**0.37%–40% strain** 9.8%–29.4 mN 300 mbar 1–8 cm	0.001–1 Hz	n/a	0.11–24 h	1–56 days	30
Shear	n/a	**2%–12% strain** ±25 °	1 Hz	n/a	0.11–1 h	3–24 days	3
Tension	5% strain	**0.5%–40% strain** ±15 °	0.0028–2 Hz	n/a	0.5–24 h	1–21 days	18
Fluid‐mediated stimulation							
Hydrostatic pressure	0.1–0.5 MPa	0.1–10 MPa	0.0014–1 Hz	n/a	1–8 h	1–56 days	15
Fluid‐induced shear	**0–1.6 Pa** 830–3800 Re	**0.003–1.64 Pa** 370–2900 Re	0.25–3.33 Hz	0.005–12 mL/min	2–24 h	1–56 days	17
Combined stimulation							
Compression and shear	5%–10% strain	**3%–20% strain (C), ±25° (S)** 15% strain (C), 10% strain (S) 5%–20% strain (C), 2.5–10 mm/s (S) 300 mbar (C), 350 mbar (S)	0.25–1 Hz	n/a	0.5–24 h	2–28 days	13
Compression and fluid‐induced shear	2% strain 0.5 N	5% strain 19.5 N	0.3–1 Hz	0.5–3 mL/min	1–24 h	14–42 days	2
Tension and fluid‐induced shear	n/a	0.085%–0.34% strain (C), 0.25–2 mm/s (S)	0.125–1 Hz	n/a	18 h	1 day	1
Hydrostatic pressure and osmotic pressure	n/a	0.5 MPa (HP), 320–450 mOsm (OP)	0.5 Hz	n/a	24 h	3–7 days	1
Compression, shear and fluid‐induced shear	n/a	3% strain (C), 5.7 mm/s (S)	n/a	12.18 mL/min	0.5 h	15 days	1
Other types of stimulation							
Centrifugal force	n/a	**10–30 000 G** 10°/s^2^	250	n/a	0.5–24 h	6–56 days	4
Ultrasonic vibration	n/a	30–150 mW/cm^2^ Ispta 1–1.5 MHz	0.33–1000 Hz	n/a	0.17–0.33 h	7–42 days	3

*Note*: the most frequently reported ranges and units are indicated in bold (if several are reported).

^a^ Multiple units are presented for the corresponding stimulation modality.

^b^ Study duration includes pre‐culture time, loading time, and resting time.

^c^ Abbreviation: C, compression; S, shear; HP, hydrostatic pressure; OP, osmotic pressure.

#### 3.3.2 Cell Source, Tissue Model, and Scaffold Design

Summary of key considerations in cartilage tissue engineering, such as cell source, tissue models, and scaffold designs, are summarized in Table 2.

**TABLE 2:** Summary of cell sources, tissue models, and scaffold designs.

Key considerations	Sub‐category	Details	Study Count
Cell source	Species	**Human**	37
		Bovine	32
		Leporine	8
		Murine	12
		Porcine	6
	Cell types	**Chondrocytes**	73
		Mesenchymal stem cells (MSC)	18
		Pluripotent stem cells (PSC)	3
		Cartilage progenitor cells (CPC)	2
		Mononuclear cells (MNC)	1
		Adipose‐derived stem cells (ASC)	1
Tissue model	2D monolayer	On a 2D substrate	26
	3D construct	**Cylindrical (0.5–10 mm diameter; 0.7–8 mm height)**	51
		Cuboidal (5–20 mm length, 4–10 mm width and 3–5 mm thickness)	6
		Spherical (2.5–8 mm diameter)	3
		Cell pellet from centrifugation	4
		Cell aggregate	1
		Other customized forms	5
Scaffold design	Hydrogel for cell encapsulation	**Natural hydrogels (agarose, alginate, collagen‐I, fibrin, gellan gum)**	23
		Hyaluronic acid hydrogels	2
		Synthetic hydrogels (GelMA, GelMA/PCL, GelMA/alginate, PEG‐ or HEMA‐based composites)	9
	Solid porous/fibrous	**Fully synthetic polymers (polyglycolic acid,** p**oly(lactic‐co‐glycolic acid), polycaprolactone, polyurethane, etc.)**	15
		Calcium‐based composites (calcium polyphosphate, collagen I/III with β‐tricalcium phosphate, Tutobone)	12
		Fibrin‐enhanced composites (polyurethane combined with fibrin or platelet‐rich plasma)	5
	Scaffold‐free	In forms of cell aggregation and pellets from centrifugation	7

*Note*: the most frequently reported information is indicated in bold (if several are reported).

#### 3.3.3 In Vitro Culture Conditions

Standard in vitro culture conditions were used in most reported studies: 37°C and 5% CO_2_. Oxygen concentration was typically not reported, with 20%–21% O_2_ reported in 4 studies, and hypoxic conditions (≤5% O_2_) used in 3 studies. Though humidity was typically not reported, 95% humidity was noted in 12 studies. Pre‐culture was another standard procedure for stabilizing tissue constructs prior to dynamic mechanical stimulation. While 20 studies did not report or implement any preculture durations, the most reported were 1–3 days (32 studies), and 4–7 days (27 studies). A smaller number of studies extended preculture to 8–14 days (11 studies) or more than 14 days, including 21, 28, 30, and 35 days (11 studies).

#### 3.3.4 Assessment Methods for Chondrogenic Outcome

Assessment methods reported for quantifying outcomes of mechanically stimulated chondrogenesis are summarized in Table 3. Metrics not considered as positive indicators for chondrogenesis, such as type‐I collagen, type‐X collagen, DNA content, and pull‐out strength for integration, were not summarized here.

**TABLE 3:** Assessment and normalization methods reported for each chondrogenic outcome.

Chondrogenic Outcome	Assessment Method	Normalization Method	Study Count
Aggrecan, type‐II collagen and *SOX9* gene expression	qPCR	**Comparative threshold cycle method with endogenous housekeeping genes** ^a^, or not normalized	55
Aggrecan and type‐II collagen protein semi‐quantification	Immunohistochemistry	Not normalized	1
GAG quantification	DMMB assay	DNA, **wet weight**, dry weight, or not normalized	35
	Blyscan assay	**DNA**, wet weight, dry weight, or not normalized	5
GAG semi‐quantification	Alcian Blue assay	Not normalized	1
	Safranin‐O staining	DNA, or not normalized	2
Collagen protein quantification	DMAB and chloramine T assay	Wet weight	1
	Hydroxyproline assay	DNA, **wet weight**, dry weight, or not normalized	17
	Sircol assay	Wet weight	1
GAG and collagen protein quantification	Radiolabel assay	**DNA**, wet weight, or not normalized	15
	ELISA	Wet weight	1
GAG and collagen protein semi‐quantification	Western blot assay	GAPDH, wet weight, or not normalized	3
	MIR spectroscopy	Not normalized	1
Compressive equilibrium modulus	Confined compression test	Not normalized	2
	Creep indentation test	Not normalized	1
	Indentation test	Not normalized	1
	Unconfined compression test	Not normalized	10
Dynamic modulus	Dynamic test	Not normalized	6

*Note*: the most frequently reported information is indicated in bold (if several are reported).

^a^ Endogenous housekeeping genes include: 18S rRNA, 28S rRNA, *B2M*, β‐actin, *GAPDH*, *RPL13A*, *RPLP0*, *SDHA*, combination of 18S + *B2M* + *HPRT1*, and combination of *HNRPH1* + *CPSF6*.

Gene expression of chondrogenic indicators such as aggrecan, type‐II collagen, and *SOX9* was mainly quantified by qPCR, using the comparative threshold cycle (ΔΔCt) method with endogenous housekeeping genes, while some studies had no normalization method at all. *GAPDH* is known to vary under certain conditions, and the combination of *HNRPH1*+*CPSF6* is not validated for general qPCR use. Only one study used immunohistochemistry without normalization to semi‐quantify aggrecan and type‐II collagen protein. Quantification of glycosaminoglycan (GAG) and collagen also used diverse assays with inconsistent normalization references. While DMMB and hydroxyproline assays were the most used, wet weight and dry weight were extensively used as the primary normalization method, which is known to be less stable compared to DNA. Western blot and radiolabel also showed variability in normalization practices. For mechanical properties, including compressive equilibrium modulus and dynamic modulus, mechanical testing typically did not describe key parameters required to compare measures from differing setups. For example, testing was conducted at different strain levels (1%–3%) and frequencies (0.1–1 Hz) for measuring the dynamic modulus, which does not permit normalization and thus, comparison between studies for assessing the mechanical performance of stimulated tissue culture.

### 3.4 Chondrogenic Effect of Mechanically Stimulated Chondrogenesis

#### 3.4.1 Overall Chondrogenic Effects for Each Stimulation Modality

**FIGURE 3:**
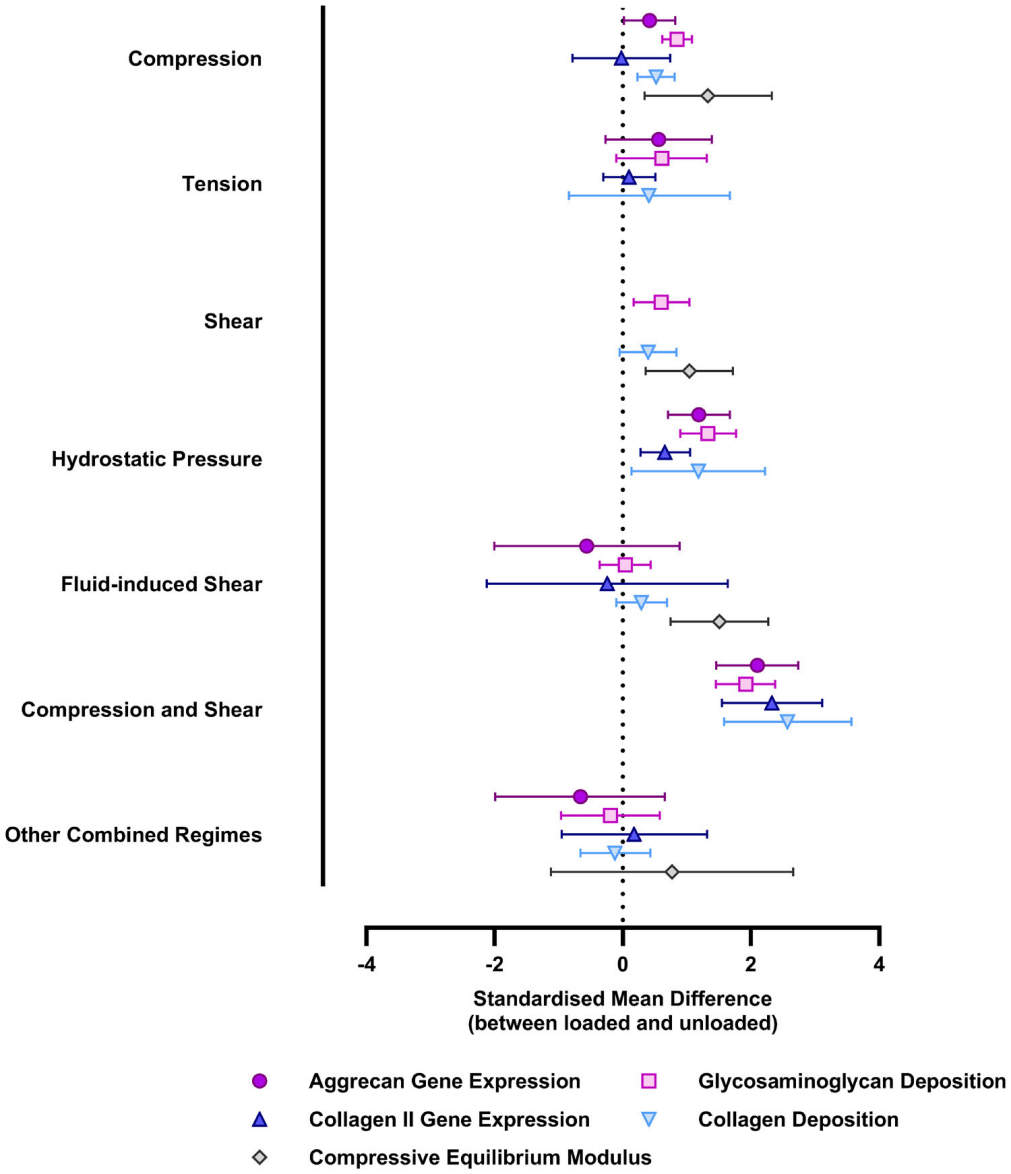
Overview of chondrogenic effects measured with aggrecan gene expression (***k*** = 136), glycosaminoglycan deposition (***k*** = 281), collagen II gene expression (***k*** = 140), collagen deposition (***k*** = 109), and compressive equilibrium modulus (***k*** = 33) for different mechanical stimulation modalities. ***k*** represents the number of distinct mechanical stimulation groups or protocols. Effect sizes are reported with standardized mean differences and 95% CIs.

Standardized effect measures for different mechanical stimulation modalities are shown in Figure 3. The corresponding meta‐analysis statistics, including number of studies, SMDs, 95% CIs and study heterogeneity, are available in Table S7.

For each mechanical stimulation modality, chondrogenic effects were assessed based on a variety of biological markers associated with cartilage matrix development, including aggrecan mRNA expression and glycosaminoglycan (GAG) content, type‐II collagen mRNA expression and collagen protein deposition. The combination of compression and shear was the most effective mechanical stimulation modality, with positive effect sizes ranging from 1.92 to 2.57. Hydrostatic pressure also showed positive effects, with effect sizes ranging from 0.66 to 1.33. Independent application of the three direct‐application stimuli generally resulted in upregulation of chondrogenic markers, effect with sizes of −0.02–0.85 for compression, 0.10–0.61 for tension, 0.40–0.60 for shear. The stimulatory effect was more pronounced for GAG deposition than for collagen protein, whereas minimal effects were observed for collagen II gene expression. Fluid‐induced shear and other combined stimulation regimes presented mixed effects.

Mechanical performance of tissue‐engineered cartilage was quantified by compressive equilibrium modulus. Compression, shear, fluid‐induced shear, and other combined regimes presented positive SMDs, suggesting dynamic mechanical stimulation generally had a beneficial effect influencing matrix material properties.

The majority of the compared groups had either substantial (50%–75%) or considerable (75%–100%) percentage heterogeneity to total variance, indicating the variability in effect sizes is not due to chance but true variation between studies such as cell source, mechanical stimulation protocol, differences in laboratories, outcome assessment method, and in vitro culture conditions.

**FIGURE 4:**
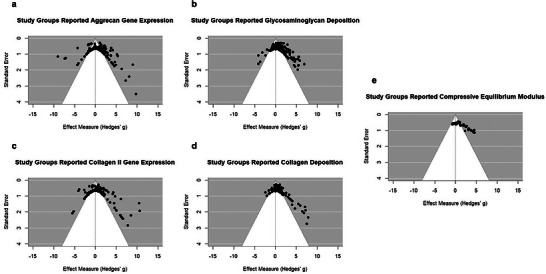
Egger's test presented in funnel plots to assess publication bias across reported outcome data: (a) aggrecan gene expression (***k*** = 136), (b) glycosaminoglycan deposition (***k*** = 281), (c) collagen II gene expression (***k*** = 140), (d) collagen deposition (***k*** = 109), and (e) compressive equilibrium modulus (***k*** = 33). ***k*** represents the number of distinct mechanical stimulation groups or protocols. Each dot represents an individual study. The white funnel region represents the expected 95% confidence assuming no bias. Symmetry of dots around the vertical dashed line (overall effect estimate) suggests low risk of publication bias, whereas asymmetry indicates potential small‐study effects or publication bias.

Egger's test was conducted to assess for publication bias or small‐study effects by evaluating funnel plot asymmetry (Figure 4). Each datapoint represents an effect size calculated from a single treatment‐control comparison within a study. Larger studies (smaller standard errors) appear toward the top of the funnel plot, and vice versa. In the absence of bias, the funnel plot should be symmetrical. Except for studies reporting aggrecan gene expression (Figure 4a), this was the case, where GAG deposition, collagen II gene expression, collagen deposition and compressive equilibrium modulus (Figure 4b‐e) all showed noticeable skewness of smaller studies toward the positive effects. Statistics of Egger's regression test confirmed the funnel plot asymmetry, with statistically significant intercepts for aggrecan gene expression (b = −1.07, *p* < 0.0001), GAG deposition (b = −0.87, *p* < 0.0001), collagen II gene expression (b = −1.47, *p* < 0.0001), collagen protein deposition (b = −1.48, *p* < 0.0001), and compressive equilibrium modulus (b = −3.11, *p* < 0.0001), suggesting the presence of small‐study effects and potential publication bias, which could contribute to inflated effect measures.

#### 3.4.2 Meta‐Regression of Effect Sizes Across Different Frequencies

Meta‐regression analysis of cartilage matrix production affected by mechanical stimulation with different amplitudes and frequencies was conducted. Detailed meta‐regression results are available in Tables S8–S17 and Figures S2–S11. The β coefficient represents the change in SMD per unit change in the predictor, which is either amplitude or frequency in this review. For amplitudes where various units were specified, direct‐application modalities, including compression, shear, and tension, were all analyzed using percent strain (%strain), while fluid‐mediated regimes, including hydrostatic pressure and fluid‐induced shear, were analyzed using MPa and ml/min, respectively.

The majority of mechanical stimulation modalities did not present statistically significant correlation (*p* > 0.05) between chondrogenic outcome and applied amplitudes or frequencies. For amplitude, (1) aggrecan gene expression was upregulated by increased amplitude (MPa) of hydrostatic pressure (β = 0.12, 95% CI [0.00, 0.25], p = 0.046); (2) GAG deposition was downregulated by increased amplitude (% strain) of tension (β = −0.08, 95% CI [−0.15, 0.00], p = 0.040) and shear (β = −0.10, 95% CI [−0.20, 0.00], p = 0.041); (3) collagen II gene expression was upregulated by increased amplitude (MPa) of hydrostatic pressure (β = 0.10, 95% CI [0.01, 0.19], p = 0.024); and (4) collagen protein deposition was downregulated by increased amplitude (% strain) of shear (β = −0.13, 95% CI [−0.22, −0.03], p = 0.011).

Comparing frequency, (1) aggrecan gene expression was downregulated by increased frequency of tension (β = −2.48, 95% CI [−4.56, −0.40], p = 0.020) and upregulated by the combination of compression and shear (β = 4.67, 95% CI [2.77, 6.58], p = 0.015); (2) GAG deposition was downregulated by increased frequency of the combination of compression and shear (β = −3.96, 95% CI [−7.84, −0.07], p = 0.046) and upregulated by other combined regimes (β = 2.84, 95% CI [0.01, 5.66], p = 0.049); (3) collagen II gene expression was downregulated by increased frequency of hydrostatic pressure (β = −2.48, 95% CI [−4.86, −0.11], p = 0.04) and upregulated by the combination of compression and shear (β = 6.01, 95% CI [2.96, 9.05], p = 0.0001); (4) collagen protein deposition downregulated by increased frequency of tension (β = −2.52, 95% CI [−4.22, −0.82], p = 0.004) and hydrostatic pressure (β = −7.16, 95% CI [−11.72, −2.59], p = 0.002); and (5) compressive equilibrium modulus was upregulated by increased frequency of other combined regimes (β = 4.02, 95% CI [1.36, 6.68], p = 0.003).

**FIGURE 5:**
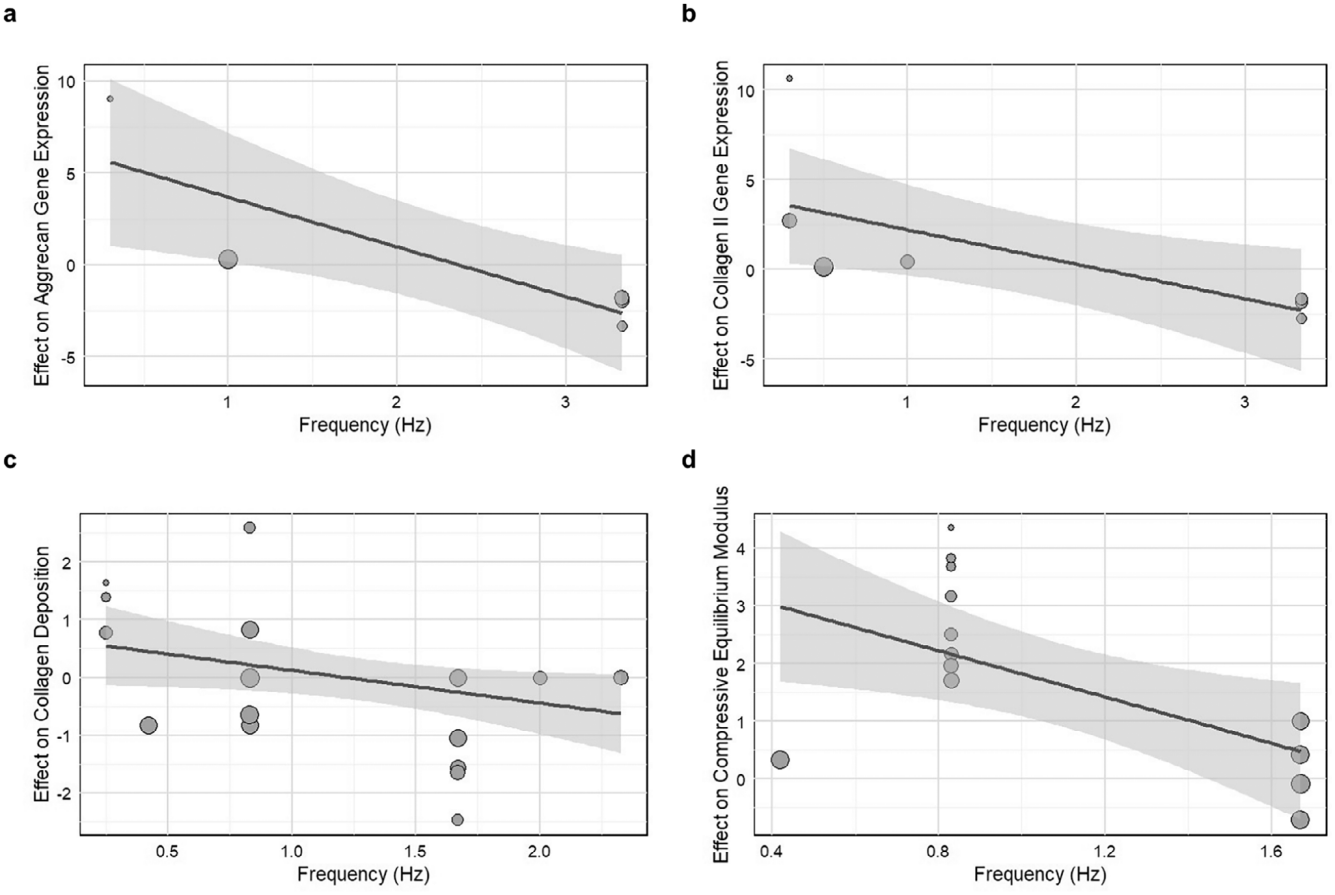
Meta‐regression presented in bubble plots, examining the effect of applied frequency (Hz) of fluid‐induced shear on cartilage tissue outcomes: (a) aggrecan gene expression, (b) collagen II gene expression, (c) collagen deposition, and (d) compressive equilibrium modulus. Stimulus mode with insufficient variations of amplitude was neglected. Each dot represents an individual study effect size with larger dots indicating greater precision (i.e. higher inverse variance). The solid line shows the fitted regression line from the meta‐regression model, representing the overall trend of the effect across frequencies, while the shaded ribbon shows the 95% confidence interval.

Particularly, stimulation with fluid‐induced shear exhibited a statistically significant negative correlation with aggrecan gene expression (β = −2.70, 95% CI [−4.61, −0.80], p = 0.005), collagen II gene expression (β = −1.92, 95% CI [−3.53, −0.32], p = 0.019), collagen deposition (β = ‐0.57, 95% CI [−1.12, −0.01], p = 0.046), and compressive equilibrium modulus (β = −2.00, 95% CI [−3.62, −0.39], p = 0.015) (Figure 5). This meta‐regression analysis suggests chondrogenic effects decreased with increasing intensity of fluid‐induced shear, typically induced under continuous flow (e.g. perfusion systems) or vortex‐based stimulation (e.g., orbital shaker, spinning flask).

## 4 Discussion

### 4.1 Standardized Effect Measures Enable Cross‐Study Quantitative Comparison

**FIGURE 6:**
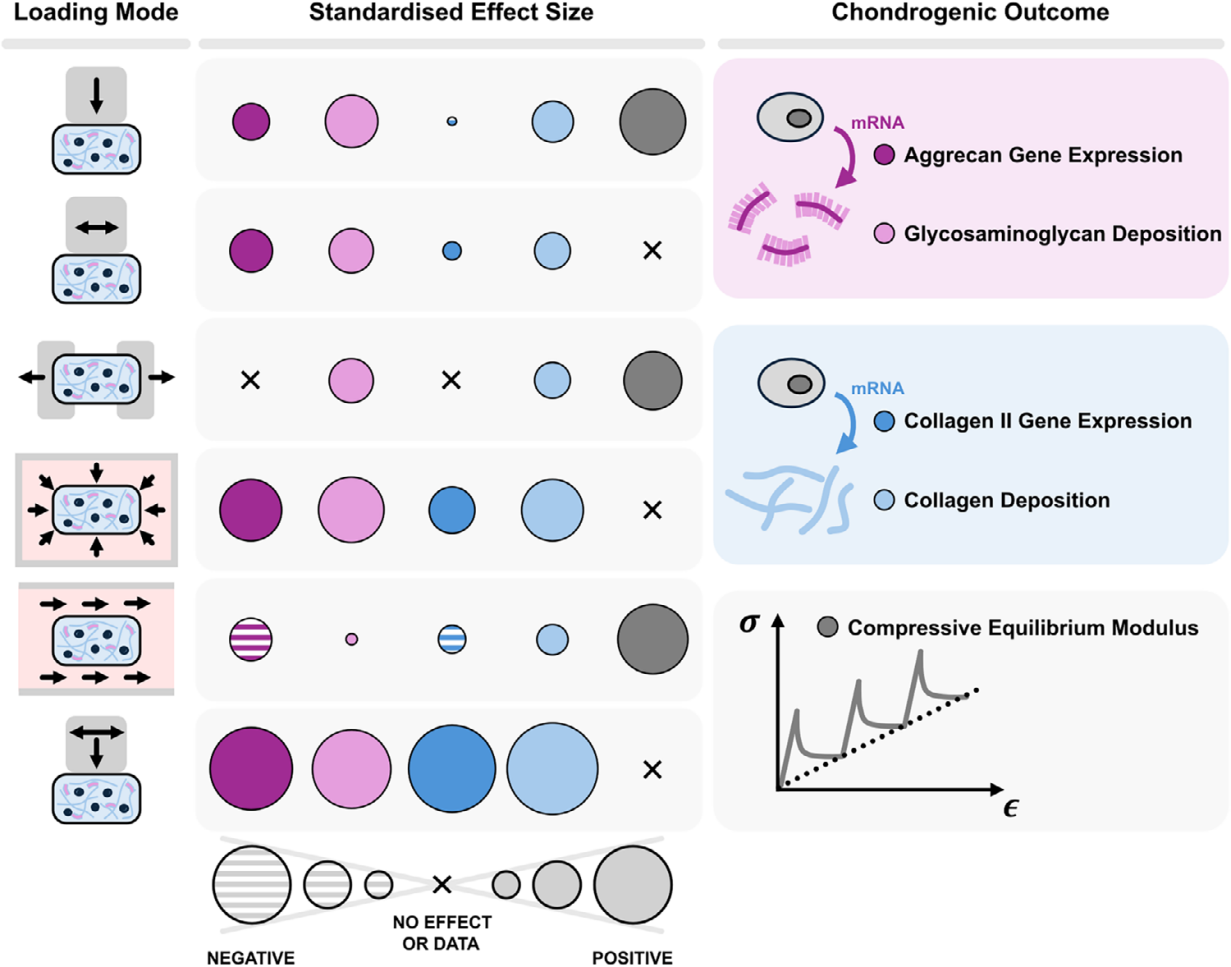
A conceptual summary of the effectiveness of conventional mechanical stimulation regimes (compression, shear, tension, hydrostatic pressure, fluid‐induced shear, and combined regimes of compression and shear) on different aspects of chondrogenic outcomes (aggrecan and collagen II gene expression, glycosaminoglycan and collagen deposition, and compressive equilibrium modulus) in in vitro cartilage tissue models. Bubble size reflects the magnitude of the standardized effect size. Solid fill indicates a positive effect, while horizontal dashed fill indicates a negative effect. Black cross marks indicate where effect sizes are either zero or not calculated due to insufficient data.

The use of standardized effect measures allowed for direct quantitative comparison of chondrogenic potential across heterogeneous studies (Figure 3). For the first time using a systematic review approach, different mechanical stimulation regimes were compared side‐by‐side (Figure 6), providing a quantitatively synthesized baseline regarding the relative effectiveness of various mechanical loading strategies. With evidence‐based starting conditions, this review reduces the initial trial‐and‐error phase of experimental design, thereby guiding the optimization of mechanical stimulation parameters for chondrogenesis in tissue engineered cartilage models.

The foundation of using standardized effect measures for quantitative comparison depends on the quality and consistency of the reported data. However, many mechanical stimulation modalities produced heterogeneity in effect estimation (Table S7), suggesting that the differences observed are likely due to true variation across studies, rather than sampling error. Egger's test (Figure 4) also revealed potential publication bias or small‐study effects in the included studies, highlighting the need to critically assess how mechanical stimulation is delivered.

To address the uncertainties identified from the meta‐analysis, the design and development of future custom‐built bioreactors can greatly benefit from practices widely used in the product design industry. Specifically, future design should consider the baseline accuracies and engineering tolerances of individually sourced components such as sensors and actuators, as well as the stack‐up variability that occurs upon system assembly. Functional verification testing against pre‐defined acceptance criteria is also a recommended practice in early‐phase development of custom prototypes for traceability. Future work ought to formalize bioreactor design and optimization guidelines specific to cartilage tissue engineering.

Furthermore, the diversity of tissue construct variables such as cell source and scaffold design observed across the included studies indicates that optimal tissue culture protocols are highly case‐specific. Future studies may adopt the Design of Experiments (DoE) framework suggested by Ladner et al. to systematically examine the synergistic interactions between variables and optimize toward a tailored protocol [129]. By ensuring hardware reliability and following DoE to navigate biological complexity, researchers can confidently benchmark against the baseline mapped in this review, significantly reducing the initial trial‐and‐error phase of experimental design.

### 4.2 Standardized Reporting is Crucial

The lack of a standardized reporting format remains a major challenge to accurately interpret and evaluate various cartilage tissue engineering protocols. Key mechanical control parameters, such as stimulation amplitude, strain rate, and frequency, were not consistently reported (Table 1) but should be a minimum requirement to allow cross‐study comparisons. This issue is evident in mechanical stimulation protocols using fluid‐induced shear, where studies often only report flow rate or frequency in Hz or rpm, with few specifying amplitudes of the applied shear pressure in pascals. Furthermore, mechanical cues expressed at the cellular level is dependent not only on the applied load at the bulk tissue level, but also the internal geometry of tissue model, such as porosity, pore size, and strut topology, which were frequently omitted across the included studies. The detailed reporting is recommended for understanding the true mechanical cues in tissue models with different microarchitectural geometry.

The same challenge applies to biological assessment (Table 3), where differences in assessment methods obfuscate the impact of mechanical stimulation on chondrogenesis. For example, chondrogenic activity for matrix development was indicated by mRNA expression (e.g., aggrecan, type‐II collagen) or matrix deposition assays (e.g., glycosaminoglycan, collagen content), each of which captures a different stage of cartilage development (Figure 6). Depending on the specificity of research aims, appropriate assessment methods must be selected to accurately capture measures of interest. Besides, current assessments mainly focus on positive hyaline biomarkers, often neglecting the negative indicators of fibrocartilage (e.g., collagen type I) or hypertrophy (e.g., collagen type X), which should be incorporated to ensure phenotypic stability in future studies. Moreover, there is often a disproportionate focus on biological outcomes (e.g., gene or protein quantification) compared to functional performance (e.g., material modulus). As highlighted by Guilak et al., a comprehensive evaluation of engineered cartilage tissue requires both biological and functional assessment to determine its clinical relevance and mechanical competence [8]. A checklist of recommended reporting items, including tissue construct variables, culture conditions, mechanical stimulation parameters, and outcome assessments, were compiled as detailed in Table S18.

In addition, the lack of a dedicated quality assessment tool tailored for in vitro mechanobiology research brings challenges. Common criteria used for clinical reviews are not applicable to in vitro studies. A standardized quality assessment framework, as presented here with the modified QUIN tool, will be valuable for improving consistency in future systematic reviews.

### 4.3 True Measure of Stimuli Remains Undefined

Control parameters specified at the bioreactor level do not indicate the true measure of stimuli in the sub‐tissue space. Tissue‐engineered cartilage constructs undergoing mechanically stimulated culture could inadvertently receive similar stimuli given two distinct sets of control parameters. For constructs undergoing fluid‐shear stimulation in spinner flasks, a similar hydrodynamic environment, characterized by Reynolds number, power dissipation, eddy size and velocity can be achieved with varying sets of angular velocity and stirring bar length [46]. Similarly, position‐controlled stimuli such as compression and tension are often controlled by percent strain, but the actual strain measured in the tissue construct can be different due to material heterogeneity [30].

Cells within tissue‐engineered cartilage construct could respond differently depending on the magnitude of the received mechanical signals. For example, physiological compressive strain (∼10%) promotes anabolic signaling, while excessive compressive strain (>50%) has been linked to catabolic responses through the activation of mechanosensitive channels such as TPRV4 and PIEZO1 [82, 130]. Without precise knowledge of the true mechanical load cells experienced at the sub‐tissue level, it is difficult to determine whether an observed response is reflected from physiological stimulation or mechanical overload. Future work should focus on characterizing mechanical microenvironments at sub‐tissue scale, utilizing analytical solutions for 2D culture [131] and multiscale finite element modelling for complex 3D constructs [94] for instance, to inform local mechanical stimulus experienced by cells and thereby directly control mechanobiological outcomes of cartilage tissue engineering in a reproducible manner. Ultimately, integrating multiscale in silico models with real‐time sensor feedback could enable the digital twinning of bioreactor systems. While technically ambitious, such closed‐loop frameworks would allow researchers to dynamically optimize mechanical stimulation parameters and scaffold designs in parallel with physical experiments, providing a powerful platform for rapid iteration and accelerated clinical translation in cartilage tissue engineering.

### 4.4 Limitations

Although the search strategy was systematically developed and PRISMA‐compliant, it is possible that all relevant publications were not captured. Articles that did not explicitly mention keywords related to “mechanical stimulation” were excluded, which potentially limits a completely comprehensive summary. Additionally, using SMD to calculate the relative effect sizes assume that the effects of biochemical stimuli, cell source, scaffold design, and culture environment (e.g. pH) are random variance. While a random‐effect model was used during subgroup analysis to handle between‐study variance, combining heterogeneous studies remains an inherent limitation. Consequently, the pooled meta‐analytical results, including the meta‐regression, could still be overly generalized.

## 5 Conclusions

This systematic review highlights the value of using standardized effect measures to enable cross‐study comparison for mechanical stimulation protocols in cartilage tissue engineering, despite methodological heterogeneity. Direct application of compression and shear was found to be the most effective mechanical stimulation modality, indicating complex loading patterns are potentially more beneficial to induce cartilage mechanoadaptation. Loading dynamics and magnitude of mechanical stimulation were correlated with chondrogenic outcomes, especially for fluid‐induced shear, which was found to have more negative effects with increasing stimulation frequency. To define an optimal mechanobiological environment for cartilage development, control parameters of mechanical stimulation need to be rigorously optimized in a standardized environment. Addressing existing gaps such as inconsistent reporting, variations in outcome assessment methods, and lack of sub‐tissue stimulus measurement is critical for facilitating bioreactor development, improving research reproducibility, and guiding future improvement of mechanically stimulated cartilage tissue engineering.

## Author Contributions

**Jiaqi K. Shen**: Conceptualization, Data curation, Formal analysis, Investigation, Methodology, Project administration, Validation, Visualization, Writing – original draft. **Tony B. Huang**: Data curation, Investigation, Validation, Writing – review & editing. **Catherine E. Davey**: Methodology, Validation (statistics), Writing – review & editing. **Elias Salzer**: Validation (cartilage mechanics), Writing – review & editing. Gerjo Van Osch: Validation (cartilage tissue engineering), Writing – review & editing. **Sandra J. Shefelbine**: Validation (cartilage tissue mechanics), Writing – review & editing. **Marcus G. Pandy**: Validation (cartilage mechanics), Supervision, Writing – review & editing. **Kathryn S. Stok**: Conceptualization, Funding acquisition, Project administration, Resources, Supervision, Validation, Writing – review & editing.

## Funding

This project is supported by the Australian Research Council Discovery Project funding scheme (DP240102160), and Jiaqi K. Shen and Tony B. Huang are recipients of Melbourne Research Scholarship from the University of Melbourne. Elias Salzer and Gerjo van Osch are partly financed by the Dutch Research Council (NWO) through the LoaD NWA‐ORC research programme (NWA1389.20.009).

## Conflicts of Interest

The authors declare no conflicts of interest.

## Supporting information




**Supporting File**: adhm71309‐sup‐0001‐SuppMat.docx.

## Data Availability

The data that support the findings of this study are available from the corresponding author upon reasonable request.
