# Supplementary material for: Standardized Effect Measures Informing Next‐Generation Strategies for Mechanical Stimulation in Cartilage Tissue Engineering
Source: Adv Healthc Mater. 2026 Jun 3;15(26):e71309. doi: 10.1002/adhm.71309 (PMC13356526; doi:10.1002/adhm.71309)
Supplement: Supplementary file 1 — Supporting File: adhm71309‐sup‐0001‐SuppMat.docx. [file ADHM-15-0-s001.docx]

# Supplementary Data

## Detailed search strategies

Table 1. Detailed search strategies and query strings used in Scopus.

| Line | Search Terms and Conditions |  |
| --- | --- | --- |
| #1 | ( articular OR joint OR hyaline ) AND cartilage* |  |
| #2 | "in vitro" |  |
| #3 | #1 AND #2 |  |
| #4 | ( mechan* OR physi* OR biophysi* ) W/2 ( stimul* OR load* OR stress* OR pressure* OR force* ) |  |
| #5 | #1 AND #2 AND #4 |  |
| #6 | osteogen* |  |
| #7 | #1 AND #2 AND #4 AND NOT #6 |  |
| #8 | limit 7 to (English language and journal article) |  |
|  | Search location is limited to title, abstract, and keyword |  |
| Query Strings:  (TITLE-ABS-KEY(( articular OR joint OR hyaline ) AND cartilage*) AND TITLE-ABS-KEY("in vitro") AND TITLE-ABS-KEY(( mechan* OR physi* OR biophysi* ) W/2 ( stimul* OR load* OR stress* OR pressure* OR force* )) AND NOT TITLE-ABS-KEY(osteogen*)) AND ( LIMIT-TO ( SRCTYPE,"j" ) ) AND ( LIMIT-TO ( DOCTYPE,"ar" ) ) AND ( LIMIT-TO ( LANGUAGE,"English" ) ) | | |

Table 2. Detailed search strategies and query strings used in MEDLINE via Ovid.

| Line | Search Terms and Conditions |
| --- | --- |
| #1 | (( articular OR joint OR hyaline ) AND cartilage*) |
| #2 | "in vitro" |
| #3 | #1 AND #2 |
| #4 | ( mechan* OR physi* OR biophysi* ) ADJ2 ( stimul* OR load* OR stress* OR pressure* OR force* ) |
| #5 | #1 AND #2 AND #4 |
| #6 | osteogen* |
| #7 | #1 AND #2 AND #4 AND NOT #6 |
| #8 | limit 7 to (English language and journal article) |
| Query Strings:  (((articular or joint or hyaline) and cartilage* and ("in vitro") and ((mechan* or physi* or biophysi*) adj2 (stimul* or load* or stress* or pressure* or force*))) not osteogen*).mp.  limit to (english language and journal article) | |

Table 3. Detailed search strategies and query strings used in EMBASE via Ovid.

| Line | Search Terms and Conditions |
| --- | --- |
| #1 | ( articular OR joint OR hyaline ) AND cartilage* |
| #2 | "in vitro" |
| #3 | #1 AND #2 |
| #4 | ( mechan* OR physi* OR biophysi* ) ADJ2 ( stimul* OR load* OR stress* OR pressure* OR force* ) |
| #5 | #1 AND #2 AND #4 |
| #6 | osteogen* |
| #7 | #1 AND #2 AND #4 AND NOT #6 |
| #8 | limit 7 to (English language and journal article) |
|  | Search location is limited to title, abstract, and keyword |
| Query Strings:  (((articular or joint or hyaline) and cartilage* and ("in-vitro") and ((mechan* or physi* or biophysi*) adj2 (stimul* or load* or stress* or pressure* or force*))) not osteogen*).mp.  limit to (english and article and journal) | |

Table 4. Detailed search strategies and query strings used in Ei Compendex via Engineering Village.

| Line | Search Terms and Conditions |
| --- | --- |
| #1 | ( articular OR joint OR hyaline ) AND cartilage* |
| #2 | "in vitro" |
| #3 | #1 AND #2 |
| #4 | (mechan* NEAR/2 stimul* OR mechan* NEAR/2 load* OR mechan* NEAR/2 stress* OR mechan* NEAR/2 pressure* OR mechan* NEAR/2 force*) OR (physi* NEAR/2 stimul* OR physi* NEAR/2 load* OR physi* NEAR/2 stress* OR physi* NEAR/2 pressure* OR physi* NEAR/2 force*) OR (biophysi* NEAR/2 stimul* OR biophysi* NEAR/2 load* OR biophysi* NEAR/2 stress* OR biophysi* NEAR/2 pressure* OR biophysi* NEAR/2 force*) |
| #5 | #1 AND #2 AND #4 |
| #6 | osteogen* |
| #7 | #1 AND #2 AND #4 AND NOT #6 |
| #8 | limit 7 to (English language and journal article) |
|  | Search location is Subject/Title/Abstract |
| Query Strings:  (((((( articular OR joint OR hyaline ) AND cartilage*) WN KY) AND (("in vitro") WN KY)) AND (((mechan* NEAR/2 stimul* OR mechan* NEAR/2 load* OR mechan* NEAR/2 stress* OR mechan* NEAR/2 pressure* OR mechan* NEAR/2 force*) OR (physi* NEAR/2 stimul* OR physi* NEAR/2 load* OR physi* NEAR/2 stress* OR physi* NEAR/2 pressure* OR physi* NEAR/2 force*) OR (biophysi* NEAR/2 stimul* OR biophysi* NEAR/2 load* OR biophysi* NEAR/2 stress* OR biophysi* NEAR/2 pressure* OR biophysi* NEAR/2 force*)) WN KY)) NOT ((osteogen*) WN KY)) AND (JA WN DT) AND (English WN LA) | |

## Data items extracted from included studies

Table 5. Data items extracted from included studies.

| Category | Data Item |
| --- | --- |
| Literature citation details | Author name |
|  | Journal name |
|  | Year of publication |
|  | Title |
|  | DOI |
|  | Corresponding author |
|  | Email address |
| Additional information | Study objective |
|  | Commercial or in-house bioreactor? |
|  | Availability of source data |
| Cell source | 3D or 2D culture |
|  | Cell type |
|  | Seeding density |
|  | Donor type |
|  | Donor age |
|  | Anatomical location |
|  | Healthy or diseased cells |
| Incubation environment | Oxygen supply |
|  | CO_2_ supply |
|  | Temperature |
|  | Humidity |
| *In-vitro* culture protocol | Growth medium |
|  | Serum usage |
|  | Antibiotic usage |
|  | Transforming growth factor usage |
|  | Hyaluronic acid usage |
|  | Supplement |
|  | Pre-construct cell passage |
|  | Post-construct preculture time |
|  | Scaffold shape |
|  | Construct design |
|  | Scaffold material |
|  | Construct dimension |
| Control parameters of mechanical stimulation | Stimulation modality |
|  | Loading direction |
|  | Loading profile |
|  | Frequency |
|  | Preload |
|  | Amplitude |
|  | Strain rate |
|  | Shear rate |
|  | Flow rate |
|  | Stimulation duration |
|  | Duty cycle |
| Chondrogenic outcome data  (Details such as timepoints, unit of measurement, normalisation method and assessment method are collected for each data item) | *ACAN* gene expression |
|  | *COL2* gene expression |
|  | *SOX9* gene expression |
|  | GAG quantification |
|  | Collagen quantification |
| Mechanical evaluation data | Equilibrium modulus |
|  | Dynamic modulus |

## Details of study characteristics

Table 6. Study characteristics for each included study.

| Reference | Reference Number | Mechanical Stimulation Protocol | Cell Species | Cell Source | Tissue Scaffold | Preculture Time |
| --- | --- | --- | --- | --- | --- | --- |
| Carroll et al. (2014) | 40 | HP 10MPa 1Hz (MSC) w5 | porcine | MSC | cylindrical | n/a |
| Carroll et al. (2014) | 40 | HP 10MPa 1Hz (PSC) w5 | porcine | PSC | cylindrical | n/a |
| Chai et al. (2010) | 41 | DC 9.5%strain 1Hz d1 | bovine | chondrocyte | cylindrical | 18h |
| Chariyev-Prinz et al. (2023) | 42 | HP 2MPa 1Hz (LD) w1 | human | MSC | n/a | 21d |
| Chariyev-Prinz et al. (2023) | 42 | HP 2MPa 1Hz (HD) w1 | human | MSC | n/a | 21d |
| Chariyev-Prinz et al. (2023) | 42 | HP 2MPa 1Hz (TGF/LD) w1 | human | MSC | n/a | 7d |
| Chariyev-Prinz et al. (2023) | 42 | HP 2MPa 1Hz (TGF/HD) w1 | human | MSC | n/a | 7d |
| Chariyev-Prinz et al. (2023) | 42 | HP 2MPa 1Hz (TGF/LD) w2 | human | MSC | n/a | 7d |
| Chariyev-Prinz et al. (2023) | 42 | HP 2MPa 1Hz (TGF/HD) w2 | human | MSC | n/a | 7d |
| Chen et al. (2017) | 43 | HP 5MPa w8 | porcine | chondrocyte | cylindrical | 28d |
| Chen et al. (2017) | 43 | FIS 0.5Hz w8 | porcine | chondrocyte | cylindrical | 28d |
| Chen et al. (2017) | 43 | CF 100G w8 | porcine | chondrocyte | cylindrical | 28d |
| Chen et al. (2023) | 44 | USVB 30mW/cm^2Ispta 0.33Hz w1 | human | MSC | n/a | 1d |
| Chen et al. (2023) | 44 | USVB 50mW/cm^2Ispta 0.33Hz w1 | human | MSC | n/a | 1d |
| Chen et al. (2023) | 44 | USVB 70mW/cm^2Ispta 0.33Hz w1 | human | MSC | n/a | 1d |
| Chen et al. (2023) | 44 | USVB 100mW/cm^2Ispta 0.33Hz w1 | human | MSC | n/a | 1d |
| Chen et al. (2023) | 44 | USVB 150mW/cm^2Ispta 0.33Hz w1 | human | MSC | n/a | 1d |
| Chung et al. (2008) | 45 | DC 15%strain 1Hz d1 | porcine | chondrocyte | cylindrical | 5d |
| Chung et al. (2008) | 45 | DC 15%strain 1Hz d5 | porcine | chondrocyte | cylindrical | 5d |
| Correia et al. (2012) | 46 | HP 5MPa 0.5Hz w4 | human | ASC | cylindrical | n/a |
| Correia et al. (2012) | 46 | HP 0.4MPa 0.5Hz w4 | human | ASC | cylindrical | n/a |
| Das et al. (2008) | 47 | DT 0.5%strain 0.5Hz (P1) d3 | human | chondrocyte | n/a | 5d |
| Das et al. (2008) | 47 | DT 0.5%strain 0.5Hz (P3) d3 | human | chondrocyte | n/a | 5d |
| Das et al. (2008) | 47 | DT 3%strain 0.5Hz (P1) d3 | human | chondrocyte | n/a | 5d |
| Das et al. (2008) | 47 | DT 3%strain 0.5Hz (P3) d3 | human | chondrocyte | n/a | 5d |
| Davisson et al. (2002) | 48 | DC 15%strain 0.001Hz d1 | bovine | chondrocyte | cylindrical | 21d |
| Davisson et al. (2002) | 48 | DC 15%strain 0.1Hz d1 | bovine | chondrocyte | cylindrical | 21d |
| Davisson et al. (2002) | 48 | DC 55%strain 0.001Hz d1 | bovine | chondrocyte | cylindrical | 21d |
| Davisson et al. (2002) | 48 | DC 55%strain 0.1Hz d1 | bovine | chondrocyte | cylindrical | 21d |
| DeCroos et al. (2006) | 49 | DC 1.4%strain 1Hz d1 | bovine | chondrocyte | cylindrical | 3d |
| DiFederico et al. (2017) | 50 | DC 15%strain 1Hz (1d pre) d2 | bovine | chondrocyte | cylindrical | 1/6d |
| DiFederico et al. (2017) | 50 | DC 15%strain 1Hz (1d pre; 40min/d) d2 | bovine | chondrocyte | cylindrical | 1/6d |
| DiFederico et al. (2017) | 50 | DC+DS 15%strain 10%strain 1Hz (1d pre) d2 | bovine | chondrocyte | cylindrical | 1/6d |
| DiFederico et al. (2017) | 50 | DC+DS 15%strain 10%strain 1Hz (1d pre; 40min/d) d2 | bovine | chondrocyte | cylindrical | 1/6d |
| DiFederico et al. (2017) | 50 | DC 15%strain 1Hz (3d pre) d2 | bovine | chondrocyte | cylindrical | 1/6d |
| DiFederico et al. (2017) | 50 | DC 15%strain 1Hz (3d pre; 40min/d) d2 | bovine | chondrocyte | cylindrical | 1/6d |
| DiFederico et al. (2017) | 50 | DC+DS 15%strain 10%strain 1Hz (3d pre) d2 | bovine | chondrocyte | cylindrical | 1/6d |
| DiFederico et al. (2017) | 50 | DC+DS 15%strain 10%strain 1Hz (3d pre; 40min/d) d2 | bovine | chondrocyte | cylindrical | 1/6d |
| Fukuda et al. (1997) | 51 | DT 5%strain 0.0028Hz d1 | bovine | chondrocyte | n/a | 4d |
| Fukuda et al. (1997) | 51 | DT 17%strain 0.17Hz d1 | bovine | chondrocyte | n/a | 4d |
| Gassner et al. (1999) | 52 | DT 20%strain 0.05Hz d2 | leporine | chondrocyte | n/a | 3d |
| Gassner et al. (1999) | 52 | DT 20%strain 0.05Hz (IL1b) d2 | leporine | chondrocyte | n/a | 3d |
| Gassner et al. (1999) | 52 | DT 20%strain 0.05Hz d3 | leporine | chondrocyte | n/a | 3d |
| Gassner et al. (1999) | 52 | DT 20%strain 0.05Hz (IL1b) d3 | leporine | chondrocyte | n/a | 3d |
| Gooch et al. (2001) | 53 | FIS 1200Re 1.67Hz w2 | bovine | chondrocyte | cylindrical | 3d |
| Gooch et al. (2001) | 53 | FIS 3300Re 2Hz w2 | bovine | chondrocyte | cylindrical | 3d |
| Gooch et al. (2001) | 53 | FIS 6700Re 2.33Hz w2 | bovine | chondrocyte | cylindrical | 3d |
| Gooch et al. (2001) | 53 | FIS 1200Re 1.67Hz w4 | bovine | chondrocyte | cylindrical | 3d |
| Gooch et al. (2001) | 53 | FIS 3300Re 2Hz w4 | bovine | chondrocyte | cylindrical | 3d |
| Gooch et al. (2001) | 53 | FIS 6700Re 2.33Hz w4 | bovine | chondrocyte | cylindrical | 3d |
| Gooch et al. (2001) | 53 | FIS 1200Re 1.67Hz w6 | bovine | chondrocyte | cylindrical | 3d |
| Gooch et al. (2001) | 53 | FIS 3300Re 2Hz w6 | bovine | chondrocyte | cylindrical | 3d |
| Gooch et al. (2001) | 53 | FIS 6700Re 2.33Hz w6 | bovine | chondrocyte | cylindrical | 3d |
| Guo et al. (2016) | 54 | FIS 1ml/min d2 | human | MSC | spherical | n/a |
| Guo et al. (2016) | 54 | FIS 1ml/min w1 | human | MSC | spherical | n/a |
| Guo et al. (2016) | 54 | FIS 1ml/min w2 | human | MSC | spherical | n/a |
| Guo et al. (2016) | 54 | FIS 1ml/min w3 | human | MSC | spherical | n/a |
| Guo et al. (2024) | 55 | HP 5MPa 1Hz w2 | human | chondrocyte | cylindrical | 14d |
| Haasper et al. (2008) | 56 | DC 10%strain 0.5Hz w1 | human | MSC | cylindrical | 1d |
| Haasper et al. (2008) | 56 | FIS 12ml/min w1 | human | MSC | cylindrical | 1d |
| Halbwirth et al. (2015) | 57 | DC 2.5%strain 1Hz d4 | human | chondrocyte | cylindrical | 12-16d |
| Hanifi et al. (2017) | 58 | USVB 30mW/cm^2Ispta 1000Hz w3 | bovine | chondrocyte | cylindrical | n/a |
| Hanifi et al. (2017) | 58 | USVB 30mW/cm^2Ispta 1000Hz w6 | bovine | chondrocyte | cylindrical | n/a |
| Hao et al. (2021) | 59 | DC 20%strain 1Hz w2 | murine | chondrocyte | rounded cuboidal | 4h |
| Hao et al. (2021) | 59 | DC+DS 20%strain 5mm/s 0.5Hz w2 | murine | chondrocyte | rounded cuboidal | 4h |
| Hao et al. (2021) | 59 | DC+DS 20%strain 2.5mm/s 0.25Hz w2 | murine | chondrocyte | rounded cuboidal | 4h |
| Hao et al. (2021) | 59 | DC+DS 10%strain 10mm/s 1Hz w2 | murine | chondrocyte | rounded cuboidal | 4h |
| Hao et al. (2021) | 59 | DC+DS 5%strain 10mm/s 1Hz w2 | murine | chondrocyte | rounded cuboidal | 4h |
| Hao et al. (2021) | 59 | DC+DS 20%strain 10mm/s 1Hz w2 | murine | chondrocyte | rounded cuboidal | 4h |
| Hao et al. (2021) | 59 | DC 20%strain 1Hz w4 | murine | chondrocyte | rounded cuboidal | 4h |
| Hao et al. (2021) | 59 | DC+DS 20%strain 5mm/s 0.5Hz w4 | murine | chondrocyte | rounded cuboidal | 4h |
| Hao et al. (2021) | 59 | DC+DS 20%strain 2.5mm/s 0.25Hz w4 | murine | chondrocyte | rounded cuboidal | 4h |
| Hao et al. (2021) | 59 | DC+DS 10%strain 10mm/s 1Hz w4 | murine | chondrocyte | rounded cuboidal | 4h |
| Hao et al. (2021) | 59 | DC+DS 5%strain 10mm/s 1Hz w4 | murine | chondrocyte | rounded cuboidal | 4h |
| Hao et al. (2021) | 59 | DC+DS 20%strain 10mm/s 1Hz w4 | murine | chondrocyte | rounded cuboidal | 4h |
| Hayashi et al. (2015) | 60 | DT 3%strain 0.5Hz 3h | human | chondrocyte | n/a | n/a |
| Hayashi et al. (2015) | 60 | DT 5%strain 0.5Hz 3h | human | chondrocyte | n/a | n/a |
| Hayashi et al. (2015) | 60 | DT 8%strain 0.5Hz 3h | human | chondrocyte | n/a | n/a |
| Hayashi et al. (2015) | 60 | DT 10%strain 0.5Hz 3h | human | chondrocyte | n/a | n/a |
| Heidenberger et al. (2025) | 61 | FIS 1Hz (IL1b) d3 | equine | chondrocyte | cylindrical | 7d |
| Hilz et al. (2014) | 62 | DC+DS 15%strain 25° 1Hz w2 | bovine | chondrocyte | cylindrical | 7d |
| Hsu et al. (2006) | 63 | USVB 67mW/cm^2Ispta 100Hz w1 | human | chondrocyte | cylindrical | 1d |
| Hsu et al. (2006) | 63 | FIS 0.003Pa 0.25Hz w1 | human | chondrocyte | cylindrical | n/a |
| Hsu et al. (2006) | 63 | USVB 67mW/cm^2Ispta 100Hz w4 | human | chondrocyte | cylindrical | 1d |
| Hsu et al. (2006) | 63 | FIS 0.003Pa 0.25Hz w4 | human | chondrocyte | cylindrical | n/a |
| Hsu et al. (2006) | 63 | USVB 67mW/cm^2Ispta 100Hz w6 | human | chondrocyte | cylindrical | 1d |
| Hsu et al. (2006) | 63 | FIS 0.003Pa 0.25Hz w6 | human | chondrocyte | cylindrical | n/a |
| Iimoto et al. (2005) | 64 | DT 7%strain 0.5Hz 18h | murine | chondrocyte | n/a | 6d |
| Ikenoue et al. (2003) | 65 | HP 1MPa 1Hz d1 | human | chondrocyte | n/a | 1d |
| Ikenoue et al. (2003) | 65 | HP 1MPa 1Hz d4 | human | chondrocyte | n/a | 1d |
| Ikenoue et al. (2003) | 65 | HP 5MPa 1Hz d1 | human | chondrocyte | n/a | 1d |
| Ikenoue et al. (2003) | 65 | HP 5MPa 1Hz d4 | human | chondrocyte | n/a | 1d |
| Ikenoue et al. (2003) | 65 | HP 10MPa 1Hz d1 | human | chondrocyte | n/a | 1d |
| Ikenoue et al. (2003) | 65 | HP 10MPa 1Hz d4 | human | chondrocyte | n/a | 1d |
| Jing et al. (2020) | 66 | DT 1%strain 1Hz 2h | murine | chondrocyte | n/a | n/a |
| Jing et al. (2020) | 66 | DT 2.5%strain 1Hz 2h | murine | chondrocyte | n/a | n/a |
| Jing et al. (2020) | 66 | DT 4%strain 1Hz 2h | murine | chondrocyte | n/a | n/a |
| Jing et al. (2020) | 66 | DT 5.5%strain 1Hz 2h | murine | chondrocyte | n/a | n/a |
| Jung et al. (2008) | 67 | DC 5%strain 0.1Hz d5 | leporine | chondrocyte | cylindrical | 1d |
| Jung et al. (2008) | 67 | DC 5%strain 0.1Hz d10 | leporine | chondrocyte | cylindrical | 1d |
| Jung et al. (2008) | 67 | DC 5%strain 0.1Hz d17 | leporine | chondrocyte | cylindrical | 1d |
| Jung et al. (2008) | 67 | DC 5%strain 0.1Hz d24 | leporine | chondrocyte | cylindrical | 1d |
| Jung et al. (2009) | 68 | DC 5%strain 0.1Hz d10 | leporine | MNC | cuboidal | 4h |
| Jung et al. (2009) | 68 | DC 5%strain 0.1Hz (FG) d10 | leporine | MNC | cuboidal | 4h |
| Jung et al. (2009) | 68 | DC 5%strain 0.1Hz d10 | leporine | MNC | cuboidal | 4h |
| Jung et al. (2009) | 68 | DC 5%strain 0.1Hz (FG) d10 | leporine | MNC | cuboidal | 4h |
| Kawanishi et al. (2007) | 69 | HP 5MPa 0.5Hz d4 | bovine | chondrocyte | pellet | 3d |
| Khan et al. (2009) | 70 | FIS 0.005ml/min w1 | bovine | chondrocyte | cylindrical | 14d |
| Khan et al. (2009) | 70 | FIS 0.01ml/min w1 | bovine | chondrocyte | cylindrical | 14d |
| Ladner et al. (2025) | 71 | DC+DS 30%strain 1Hz w4 | human | MSC | cylindrical | 1d |
| Lee et al. (2002) | 72 | FIS 1.64Pa 3.33Hz (2hr/d) d0.67 | human | chondrocyte | n/a | 1d |
| Lee et al. (2002) | 72 | FIS 1.64Pa 3.33Hz (6hr/d) d1 | human | chondrocyte | n/a | 1d |
| Lee et al. (2002) | 72 | FIS 1.64Pa 3.33Hz (24hr/d) d2 | human | chondrocyte | n/a | 1d |
| Lee et al. (2003) | 73 | HP 10MPa 1Hz d1 | human | chondrocyte | n/a | n/a |
| Lee et al. (2020) | 74 | DC 40%strain 0.5Hz (8%PEG) w1 | human | MSC | cylindrical | n/a |
| Lee et al. (2020) | 74 | DC 40%strain 0.5Hz (12%PEG) w1 | human | MSC | cylindrical | n/a |
| Lee et al. (2020) | 74 | DC 40%strain 0.5Hz (8%PEG) w2 | human | MSC | cylindrical | n/a |
| Lee et al. (2020) | 74 | DC 40%strain 0.5Hz (12%PEG) w2 | human | MSC | cylindrical | n/a |
| Lee et al. (2020) | 74 | DC 40%strain 0.5Hz (8%PEG) w3 | human | MSC | cylindrical | n/a |
| Lee et al. (2020) | 74 | DC 40%strain 0.5Hz (12%PEG) w3 | human | MSC | cylindrical | n/a |
| Liang et al. (2015) | 75 | HP 0.2MPa 0.1Hz d3 | leporine | chondrocyte | n/a | n/a |
| Liang et al. (2015) | 75 | HP 0.2MPa 0.1Hz w1 | leporine | chondrocyte | n/a | n/a |
| Lima et al. (2008) | 76 | DC 15%strain 1Hz w2 | bovine | chondrocyte | cylindrical | 14d |
| Lima et al. (2008) | 76 | DC 15%strain 1Hz (IL1b) w2 | bovine | chondrocyte | cylindrical | 14d |
| Lima et al. (2008) | 76 | DC 15%strain 1Hz (IL1a) w2 | bovine | chondrocyte | cylindrical | 14d |
| Limraksasin et al. (2020) | 77 | FIS 0.5Hz d10 | murine | PSC | pellet | 4d |
| Limraksasin et al. (2020) | 77 | FIS 0.3Hz d10 | murine | PSC | pellet | 4d |
| Limraksasin et al. (2020) | 77 | FIS 0.5Hz d17 | murine | PSC | pellet | 4d |
| Limraksasin et al. (2020) | 77 | FIS 0.3Hz d17 | murine | PSC | pellet | 4d |
| Limraksasin et al. (2020) | 77 | FIS 0.5Hz d24 | murine | PSC | pellet | 4d |
| Lin et al. (2017) | 78 | DC 15%strain 1Hz w2 | murine | MSC | cylindrical | 1d |
| Liu et al. (2025) | 79 | DT 6%strain 0.5Hz d1 | murine | chondrocyte | n/a | n/a |
| Liu et al. (2025) | 79 | DT 18%strain 0.5Hz d1 | murine | chondrocyte | n/a | n/a |
| Long et al. (2001) | 80 | DT 6%strain 0.05Hz d3 | leporine | chondrocyte | n/a | 8d |
| Long et al. (2001) | 80 | DT 6%strain 0.05Hz d4 | leporine | chondrocyte | n/a | 8d |
| Lou et al. (2023) | 81 | DC 1cm 0.15Hz w8 | murine | chondrocyte | n/a | 1d |
| Lou et al. (2023) | 81 | DC 2cm 0.15Hz w8 | murine | chondrocyte | n/a | 1d |
| Lou et al. (2023) | 81 | DC 4cm 0.15Hz w8 | murine | chondrocyte | n/a | 1d |
| Lou et al. (2023) | 81 | DC 8cm 0.15Hz w8 | murine | chondrocyte | n/a | 1d |
| Lückgen et al. (2022) | 82 | DC 35%strain 1Hz (21d pre) d1 | human | chondrocyte | cylindrical | 21d |
| Lückgen et al. (2022) | 82 | DC 35%strain 1Hz (21d pre; MSC) d1 | human | MSC | cylindrical | 21d |
| Lückgen et al. (2022) | 82 | DC 35%strain 1Hz (35d pre) d1 | human | chondrocyte | cylindrical | 35d |
| Lückgen et al. (2022) | 82 | DC 35%strain 1Hz (35d pre; MSC) d1 | human | MSC | cylindrical | 35d |
| Lückgen et al. (2024) | 83 | DC 35%strain 1Hz d1 | human | MSC | cylindrical | 21d |
| Luo et al. (2015) | 84 | DC 10%strain 1Hz (MSC) w3 | porcine | MSC | cylindrical | 21d |
| Luo et al. (2015) | 84 | DC 10%strain 1Hz (PSC) w3 | porcine | PSC | cylindrical | 21d |
| Madhavan et al. (2006) | 85 | DT 3%strain 0.25Hz (4hr/d) d1 | murine | chondrocyte | n/a | 5-6d |
| Madhavan et al. (2006) | 85 | DT 3%strain 0.25Hz (16hr/d) d1 | murine | chondrocyte | n/a | 5-6d |
| Madhavan et al. (2006) | 85 | DT 3%strain 0.25Hz (20hr/d) d1 | murine | chondrocyte | n/a | 5-6d |
| Madhavan et al. (2006) | 85 | DT 3%strain 0.25Hz (24hr/d) d1 | murine | chondrocyte | n/a | 5-6d |
| Madhavan et al. (2006) | 85 | DT 3%strain 0.25Hz (8hr/d) d1 | murine | chondrocyte | n/a | 5-6d |
| Madhavan et al. (2006) | 85 | DT 3%strain 0.25Hz (12hr/d) d1 | murine | chondrocyte | n/a | 5-6d |
| Maeda et al. (2005) | 86 | CF 30000G 250Hz d3 | leporine | chondrocyte | in culture tube | 2d |
| Maeda et al. (2005) | 86 | CF 30000G 250Hz d5 | leporine | chondrocyte | in culture tube | 2d |
| Maeda et al. (2005) | 86 | CF 30000G 250Hz w1 | leporine | chondrocyte | in culture tube | 2d |
| Maeda et al. (2005) | 86 | CF 30000G 250Hz w2 | leporine | chondrocyte | in culture tube | 2d |
| Maeda et al. (2005) | 86 | CF 30000G 250Hz w3 | leporine | chondrocyte | in culture tube | 2d |
| Maeda et al. (2005) | 86 | CF 30000G 250Hz w4 | leporine | chondrocyte | in culture tube | 2d |
| Matsubara et al. (2019) | 87 | DT 5%strain 1Hz 3hr | murine | chondrocyte | cuboidal | n/a |
| Matsubara et al. (2019) | 87 | DT 10%strain 1Hz 3hr | murine | chondrocyte | cuboidal | n/a |
| Matsubara et al. (2019) | 87 | DT 16%strain 1Hz 3hr | murine | chondrocyte | cuboidal | n/a |
| Matsubara et al. (2019) | 87 | DT 5%strain 1Hz 24hr | murine | chondrocyte | cuboidal | n/a |
| Matsubara et al. (2019) | 87 | DT 10%strain 1Hz 24hr | murine | chondrocyte | cuboidal | n/a |
| Matsubara et al. (2019) | 87 | DT 16%strain 1Hz 24hr | murine | chondrocyte | cuboidal | n/a |
| Mawatari et al. (2010) | 88 | DT+FIS 0.085%strain 0.125Hz 0.25mm/s d1 | human | chondrocyte | n/a | 5-7d |
| Mawatari et al. (2010) | 88 | DT+FIS 0.085%strain 1Hz 2mm/s d1 | human | chondrocyte | n/a | 5-7d |
| Mawatari et al. (2010) | 88 | DT+FIS 0.17%strain 0.125Hz 0.25mm/s d1 | human | chondrocyte | n/a | 5-7d |
| Mawatari et al. (2010) | 88 | DT+FIS 0.34%strain 0.125Hz 0.5mm/s d1 | human | chondrocyte | n/a | 5-7d |
| Mawatari et al. (2010) | 88 | DT+FIS 0.17%strain 0.125Hz 0.5mm/s d1 | human | chondrocyte | n/a | 5-7d |
| Mhanna et al. (2013) | 89 | DT 5%strain 1Hz (4d pre) 1d | bovine | chondrocyte | n/a | 4d |
| Mhanna et al. (2013) | 89 | DT 5.8%strain 1Hz (4d pre) 1d | bovine | chondrocyte | cuboidal | 4d |
| Mhanna et al. (2013) | 89 | DT 5%strain 1Hz (1d pre) 4d | bovine | chondrocyte | n/a | 1d |
| Mhanna et al. (2013) | 89 | DT 5.8%strain 1Hz (1d pre) 4d | bovine | chondrocyte | cuboidal | 1d |
| Miyanishi et al. (2006) | 90 | HP 0.1MPa 1Hz w2 | human | MSC | pellet | 7-14d |
| Miyanishi et al. (2006) | 90 | HP 1MPa 1Hz w2 | human | MSC | pellet | 7-14d |
| Miyanishi et al. (2006) | 90 | HP 10MPa 1Hz w2 | human | MSC | pellet | 7-14d |
| Miyanishi et al. (2006) | 91 | HP 10MPa 1Hz w2 | human | MSC | pellet | 7-14d |
| Miyanishi et al. (2006) | 91 | HP 10MPa 1Hz (TGF) w2 | human | MSC | pellet | 7-14d |
| Mizuno et al. (2011) | 92 | HP+OP 0.5Mpa 320mOsm 0.5Hz (SZ) w1 | bovine | chondrocyte | tubular | n/a |
| Mizuno et al. (2011) | 92 | HP+OP 0.5Mpa 450mOsm 0.5Hz (SZ) w1 | bovine | chondrocyte | tubular | n/a |
| Mizuno et al. (2011) | 92 | HP+OP 0.5Mpa 320mOsm 0.5Hz (SZ) w1 | bovine | chondrocyte | tubular | 3d |
| Mizuno et al. (2011) | 92 | HP+OP 0.5Mpa 450mOsm 0.5Hz (SZ) w1 | bovine | chondrocyte | tubular | 3d |
| Mizuno et al. (2011) | 92 | HP+OP 0.5Mpa 320mOsm 0.5Hz (MZ) w1 | bovine | chondrocyte | tubular | n/a |
| Mizuno et al. (2011) | 92 | HP+OP 0.5Mpa 450mOsm 0.5Hz (MZ) w1 | bovine | chondrocyte | tubular | n/a |
| Mizuno et al. (2011) | 92 | HP+OP 0.5Mpa 320mOsm 0.5Hz (MZ) w1 | bovine | chondrocyte | tubular | 3d |
| Mizuno et al. (2011) | 92 | HP+OP 0.5Mpa 450mOsm 0.5Hz (MZ) w1 | bovine | chondrocyte | tubular | 3d |
| Mizuno et al. (2011) | 92 | HP+OP 0.5Mpa 320mOsm 0.5Hz (DZ) w1 | bovine | chondrocyte | tubular | n/a |
| Mizuno et al. (2011) | 92 | HP+OP 0.5Mpa 450mOsm 0.5Hz (DZ) w1 | bovine | chondrocyte | tubular | n/a |
| Mizuno et al. (2011) | 92 | HP+OP 0.5Mpa 320mOsm 0.5Hz (DZ) w1 | bovine | chondrocyte | tubular | 3d |
| Mizuno et al. (2011) | 92 | HP+OP 0.5Mpa 450mOsm 0.5Hz (DZ) w1 | bovine | chondrocyte | tubular | 3d |
| Monaco et al. (2021) | 93 | DC+DS 20%strain 25° 1Hz w1 | human | MSC | cylindrical | n/a |
| Monaco et al. (2021) | 93 | DC+DS 20%strain 25° 1Hz (HA) w1 | human | MSC | cylindrical | n/a |
| Monaco et al. (2021) | 93 | DC+DS 20%strain 25° 1Hz w2 | human | MSC | cylindrical | n/a |
| Monaco et al. (2021) | 93 | DC+DS 20%strain 25° 1Hz (HA) w2 | human | MSC | cylindrical | n/a |
| Monaco et al. (2021) | 93 | DC+DS 20%strain 25° 1Hz w3 | human | MSC | cylindrical | n/a |
| Monaco et al. (2021) | 93 | DC+DS 20%strain 25° 1Hz (HA) w3 | human | MSC | cylindrical | n/a |
| Monaco et al. (2021) | 93 | DC+DS 20%strain 25° 1Hz w4 | human | MSC | cylindrical | n/a |
| Monaco et al. (2021) | 93 | DC+DS 20%strain 25° 1Hz (HA) w4 | human | MSC | cylindrical | n/a |
| Nasrollahzadeh et al. (2022) | 94 | DC 20%strain 1Hz (32oC) d6 | human | CPC | cylindrical | 4d |
| Nasrollahzadeh et al. (2022) | 94 | DC 20%strain 1Hz (37oC) d6 | human | CPC | cylindrical | 4d |
| Nebelung et al. (2012) | 95 | DC 10%strain 0.3Hz w4 | human | chondrocyte | cylindrical | 1d |
| Paggi et al. (2022) | 96 | DC 300mbar 1Hz w1 | human | chondrocyte | cuboidal | 1d |
| Paggi et al. (2022) | 96 | DC+DS 300mbar 350mbar 0.33Hz w1 | human | chondrocyte | cuboidal | 1d |
| Pötter et al. (2021) | 97 | DC+DS 15%strain 25° 1Hz (FG) w2 | human | chondrocyte | cylindrical | 7d |
| Pötter et al. (2021) | 97 | DC+DS 15%strain 25° 1Hz (PRP) w2 | human | chondrocyte | cylindrical | 7d |
| Pötter et al. (2021) | 97 | DC+DS 15%strain 25° 1Hz (FG+PL) w2 | human | chondrocyte | cylindrical | 7d |
| Pourmohammadali et al. (2020) | 98 | DC+DS+FIS 3%strain 5.7mm/s 12.18ml/min w3 | bovine | chondrocyte | cylindrical | 10d |
| Pourmohammadali et al. (2020) | 98 | DC+DS 3%strain 5.7mm/s w3 | bovine | chondrocyte | cylindrical | 10d |
| Prittinen et al. (2019) | 99 | CF 771G (15min/d) w1 | bovine | chondrocyte | cylindrical | 1d |
| Prittinen et al. (2019) | 99 | CF 771G (45min/d) w1 | bovine | chondrocyte | cylindrical | 1d |
| Prittinen et al. (2019) | 99 | CF 771G (15min/d) w2 | bovine | chondrocyte | cylindrical | 1d |
| Prittinen et al. (2019) | 99 | CF 771G (45min/d) w2 | bovine | chondrocyte | cylindrical | 1d |
| Prittinen et al. (2019) | 99 | CF 771G (15min/d) w4 | bovine | chondrocyte | cylindrical | 1d |
| Prittinen et al. (2019) | 99 | CF 771G (45min/d) w4 | bovine | chondrocyte | cylindrical | 1d |
| Raizman et al. (2009) | 100 | DC 1.4%strain 1Hz (SMZ) d1 | bovine | chondrocyte | cylindrical | 4d |
| Raizman et al. (2009) | 100 | DC 1.4%strain 1Hz (DZ) d1 | bovine | chondrocyte | cylindrical | 4d |
| Salinas et al. (2020) | 101 | FIS 0.01Pa 0.42Hz d6 | bovine | chondrocyte | cylindrical | 7d |
| Salinas et al. (2020) | 101 | FIS 0.15Pa 0.83Hz d6 | bovine | chondrocyte | cylindrical | 7d |
| Salinas et al. (2020) | 101 | FIS 0.21Pa 0.83Hz d6 | bovine | chondrocyte | cylindrical | 7d |
| Salinas et al. (2020) | 101 | FIS 0.7Pa 1.67Hz d6 | bovine | chondrocyte | cylindrical | 7d |
| Salinas et al. (2020) | 101 | FIS 0.85Pa 1.67Hz d6 | bovine | chondrocyte | cylindrical | 7d |
| Salinas et al. (2020) | 101 | FIS 0.15Pa 0.83Hz d12 | bovine | chondrocyte | cylindrical | 7d |
| Salinas et al. (2020) | 101 | FIS 0.21Pa 0.83Hz d12 | bovine | chondrocyte | cylindrical | 7d |
| Salinas et al. (2020) | 101 | FIS 0.7Pa 1.67Hz d12 | bovine | chondrocyte | cylindrical | 7d |
| Salinas et al. (2020) | 101 | FIS 0.85Pa 1.67Hz d12 | bovine | chondrocyte | cylindrical | 7d |
| Salinas et al. (2020) | 101 | FIS 0.21Pa 0.83Hz w3 | bovine | chondrocyte | cylindrical | 7d |
| Salinas et al. (2020) | 101 | FIS 0.21Pa 0.83Hz (TGF) w3 | bovine | chondrocyte | cylindrical | 7d |
| Salinas et al. (2020) | 101 | FIS 0.21Pa 0.83Hz w7 | bovine | chondrocyte | cylindrical | 7d |
| Salinas et al. (2020) | 101 | FIS 0.21Pa 0.83Hz (TGF) w7 | bovine | chondrocyte | cylindrical | 7d |
| Salzmann et al. (2009) | 102 | DC+DS 15%strain 25° 1Hz w2 | bovine | chondrocyte | cylindrical | 7d |
| Salzmann et al. (2009) | 102 | DC+DS 15%strain 25° 1Hz w4 | bovine | chondrocyte | cylindrical | 7d |
| Salzmann et al. (2011) | 103 | DC+DS 15%strain 25° 1Hz (MF) w2 | bovine | chondrocyte | cylindrical | 7d |
| Salzmann et al. (2011) | 103 | DC+DS 15%strain 25° 1Hz (LF) w2 | bovine | chondrocyte | cylindrical | 7d |
| Salzmann et al. (2011) | 103 | DC+DS 15%strain 25° 1Hz (MT) w2 | bovine | chondrocyte | cylindrical | 7d |
| Salzmann et al. (2011) | 103 | DC+DS 15%strain 25° 1Hz (LT) w2 | bovine | chondrocyte | cylindrical | 7d |
| Salzmann et al. (2011) | 103 | DC+DS 15%strain 25° 1Hz (T) w2 | bovine | chondrocyte | cylindrical | 7d |
| Salzmann et al. (2011) | 103 | DC+DS 15%strain 25° 1Hz (P) w2 | bovine | chondrocyte | cylindrical | 7d |
| Salzmann et al. (2011) | 103 | DC+DS 15%strain 25° 1Hz (X) w2 | bovine | chondrocyte | cylindrical | 7d |
| Salzmann et al. (2011) | 103 | DC+DS 15%strain 25° 1Hz (Y) w2 | bovine | chondrocyte | cylindrical | 7d |
| Schätti et al. (2011) | 104 | DC 20%strain 1Hz w3 | human | MSC | cylindrical | 2-4d |
| Schätti et al. (2011) | 104 | DS 25° 1Hz w3 | human | MSC | cylindrical | 2-4d |
| Schätti et al. (2011) | 104 | DC+DS 20%strain 25° 1Hz w3 | human | MSC | cylindrical | 2-4d |
| Seidel et al. (2004) | 105 | DC+FIS 7%strain 0.3Hz 3ml/min d10 | bovine | chondrocyte | cylindrical | 30d |
| Seidel et al. (2004) | 105 | FIS 3ml/min d10 | bovine | chondrocyte | cylindrical | 30d |
| Seidel et al. (2004) | 105 | DC+FIS 7%strain 0.3Hz 3ml/min d17 | bovine | chondrocyte | cylindrical | 30d |
| Seidel et al. (2004) | 105 | FIS 3ml/min d17 | bovine | chondrocyte | cylindrical | 30d |
| Seidel et al. (2004) | 105 | DC+FIS 7%strain 0.3Hz 3ml/min d37 | bovine | chondrocyte | cylindrical | 30d |
| Seidel et al. (2004) | 105 | FIS 3ml/min d37 | bovine | chondrocyte | cylindrical | 30d |
| Shimazaki et al. (2006) | 106 | DC 0.37%strain 0.33Hz d1 | human | chondrocyte | n/a | n/a |
| Shimazaki et al. (2006) | 106 | DC 0.37%strain 0.33Hz (OA) d1 | human | chondrocyte | n/a | n/a |
| Shimazaki et al. (2006) | 106 | DC 0.37%strain 0.33Hz (3 hr post stim) d1 | human | chondrocyte | n/a | n/a |
| Shimazaki et al. (2006) | 106 | DC 0.37%strain 0.33Hz (3 hr post stim; OA) d1 | human | chondrocyte | n/a | n/a |
| Shimazaki et al. (2006) | 106 | DC 0.37%strain 0.33Hz (6 hr post stim) d1 | human | chondrocyte | n/a | n/a |
| Shimazaki et al. (2006) | 106 | DC 0.37%strain 0.33Hz (6 hr post stim; OA) d1 | human | chondrocyte | n/a | n/a |
| Shin et al. (2006) | 107 | HP 0.2MPa 0.0014Hz (3d/w) d1 | porcine | chondrocyte | n/a | 1d |
| Shin et al. (2006) | 107 | HP 0.2MPa 0.0014Hz (6d/w) d1 | porcine | chondrocyte | n/a | 1d |
| Shin et al. (2006) | 107 | HP 0.2MPa 0.0014Hz (3d/w) d3 | porcine | chondrocyte | n/a | 1d |
| Shin et al. (2006) | 107 | HP 0.2MPa 0.0014Hz (6d/w) d3 | porcine | chondrocyte | n/a | 1d |
| Shin et al. (2006) | 107 | HP 0.2MPa 0.0014Hz (3d/w) d5 | porcine | chondrocyte | n/a | 1d |
| Shin et al. (2006) | 107 | HP 0.2MPa 0.0014Hz (6d/w) d5 | porcine | chondrocyte | n/a | 1d |
| Shin et al. (2006) | 107 | HP 0.2MPa 0.0014Hz (3d/w) w1 | porcine | chondrocyte | n/a | 1d |
| Shin et al. (2006) | 107 | HP 0.2MPa 0.0014Hz (6d/w) w1 | porcine | chondrocyte | n/a | 1d |
| Silva et al. (2020) | 108 | FIS 0.2ml/min w3 | human | MSC | cuboidal | 14d |
| Smith et al. (1995) | 109 | FIS 2.2Pa 3.33Hz (bovine) d2 | bovine | chondrocyte | n/a | 2d |
| Smith et al. (1995) | 109 | FIS 2.2Pa 3.33Hz (human) d2 | human | chondrocyte | n/a | 2d |
| Smith et al. (1996) | 110 | HP 10MPa 1Hz (FBS-) d1 | bovine | chondrocyte | n/a | 4d |
| Smith et al. (1996) | 110 | HP 10MPa 1Hz d1 | bovine | chondrocyte | n/a | 4d |
| Smith et al. (1996) | 110 | HP 10MPa 1Hz (3%FBS) d1 | bovine | chondrocyte | n/a | 4d |
| Smith et al. (2011) | 111 | HP 10MPa 1Hz d4 | human | chondrocyte | n/a | 5-7d |
| Spiteri et al. (2010) | 112 | DC 9.8mN 1Hz d1 | bovine | chondrocyte | cylindrical | 3d |
| Stampoultzis et al. (2023) | 113 | DC 20%strain 1Hz d6 | human | chondrocyte | cylindrical | 5d |
| Tran et al. (2011) | 114 | DC+FIS 20N 1Hz 0.5ml/min w4 | porcine | chondrocyte | cylindrical | 10d |
| Tran et al. (2011) | 114 | FIS 0.5ml/min w4 | porcine | chondrocyte | hollow-cylindrical | 10d |
| Valonen et al. (2010) | 115 | FIS d1 | human | MSC | cylindrical | n/a |
| Valonen et al. (2010) | 115 | FIS w1 | human | MSC | cylindrical | n/a |
| Valonen et al. (2010) | 115 | FIS w2 | human | MSC | cylindrical | n/a |
| Valonen et al. (2010) | 115 | FIS w3 | human | MSC | cylindrical | n/a |
| Vanderploeg et al. (2008) | 116 | DT 10%strain 1Hz (SZ) d3 | bovine | chondrocyte | cuboidal | 7d |
| Vanderploeg et al. (2008) | 116 | DT 10%strain 1Hz (MZ) d3 | bovine | chondrocyte | cuboidal | 7d |
| Vanderploeg et al. (2008) | 116 | DT 10%strain 1Hz (DZ) d3 | bovine | chondrocyte | cuboidal | 7d |
| Visser et al. (2015) | 117 | DC 20%strain 1Hz w2 | human | chondrocyte | cylindrical | 14d |
| Visser et al. (2015) | 117 | DC 20%strain 1Hz (PCL) w2 | human | chondrocyte | cylindrical | 14d |
| Wakimoto et al. (2024) | 118 | DT 15%strain 1Hz (24hr/d) d1 | murine | chondrocyte | n/a | 14d |
| Wakimoto et al. (2024) | 118 | DT 15%strain 1Hz (12hr/d) d0.5 | murine | chondrocyte | n/a | 14d |
| Wakimoto et al. (2024) | 118 | DT 15%strain 0.5Hz (24hr/d) d1 | murine | chondrocyte | n/a | 14d |
| Wakimoto et al. (2024) | 118 | DT 15%strain 0.5Hz (12hr/d) d0.5 | murine | chondrocyte | n/a | 14d |
| Wakimoto et al. (2024) | 118 | DT 8%strain 1Hz (24hr/d) d1 | murine | chondrocyte | n/a | 14d |
| Wakimoto et al. (2024) | 118 | DT 8%strain 1Hz (12hr/d) d0.5 | murine | chondrocyte | n/a | 14d |
| Wakimoto et al. (2024) | 118 | DT 8%strain 0.5Hz (24hr/d) d1 | murine | chondrocyte | n/a | 14d |
| Wakimoto et al. (2024) | 118 | DT 8%strain 0.5Hz (12hr/d) d0.5 | murine | chondrocyte | n/a | 14d |
| Waldman et al. (2003) | 119 | DS 2%strain 1Hz (6.7min/d) w1 | bovine | chondrocyte | cylindrical | 28d |
| Waldman et al. (2003) | 119 | DS 6%strain 1Hz (6.7min/d) w1 | bovine | chondrocyte | cylindrical | 28d |
| Waldman et al. (2003) | 119 | DS 12%strain 1Hz (6.7min/d) w1 | bovine | chondrocyte | cylindrical | 28d |
| Waldman et al. (2003) | 119 | DS 2%strain 1Hz (33.3min/d) w1 | bovine | chondrocyte | cylindrical | 28d |
| Waldman et al. (2003) | 119 | DS 6%strain 1Hz (33.3min/d) w1 | bovine | chondrocyte | cylindrical | 28d |
| Waldman et al. (2003) | 119 | DS 12%strain 1Hz (33.3min/d) w1 | bovine | chondrocyte | cylindrical | 28d |
| Waldman et al. (2003) | 119 | DS 2%strain 1Hz (6.7min/d) w8 | bovine | chondrocyte | cylindrical | 28d |
| Waldman et al. (2003) | 120 | DC 5%strain 1Hz w4 | bovine | chondrocyte | cylindrical | 28d |
| Waldman et al. (2003) | 120 | DS 2%strain 1Hz w4 | bovine | chondrocyte | cylindrical | 28d |
| Waldman et al. (2004) | 121 | DC 5%strain 1Hz w4 | bovine | chondrocyte | cylindrical | 28d |
| Waldman et al. (2004) | 121 | DC 5%strain 1Hz w4 | bovine | chondrocyte | cylindrical | 28d |
| Waldman et al. (2006) | 122 | DC 9.8mN 1Hz d1 | bovine | chondrocyte | cylindrical | 1d |
| Waldman et al. (2006) | 122 | DC 29.4mN 1Hz d1 | bovine | chondrocyte | cylindrical | 1d |
| Waldman et al. (2006) | 122 | DC 19.6mN 1Hz d1 | bovine | chondrocyte | cylindrical | 1d |
| Waldman et al. (2006) | 122 | DC 9.8mN 1Hz 15min | bovine | chondrocyte | cylindrical | 1d |
| Waldman et al. (2006) | 122 | DC 9.8mN 1Hz 30min | bovine | chondrocyte | cylindrical | 1d |
| Waldman et al. (2006) | 122 | DC 9.8mN 1Hz 1hr | bovine | chondrocyte | cylindrical | 1d |
| Wang and Tsai (2013) | 123 | DT 40%strain 0.1Hz w1 | leporine | chondrocyte | cylindrical | 3d |
| Wang and Tsai (2013) | 123 | DT 40%strain 0.1Hz w2 | leporine | chondrocyte | cylindrical | 3d |
| Wang et al. (2013) | 124 | DC+DS 15%strain 25° 1Hz (P1) w2 | bovine | chondrocyte | cylindrical | 1d |
| Wang et al. (2013) | 124 | DC+DS 15%strain 25° 1Hz (P1) w4 | bovine | chondrocyte | cylindrical | 1d |
| Wang et al. (2013) | 124 | DC+DS 15%strain 25° 1Hz (P3) w2 | bovine | chondrocyte | cylindrical | 1d |
| Wang et al. (2013) | 124 | DC+DS 15%strain 25° 1Hz (P3) w4 | bovine | chondrocyte | cylindrical | 1d |
| Wernike et al. (2008) | 125 | DC+DS 30%strain 25° 0.5Hz (21%O2) d2 | bovine | chondrocyte | cylindrical | 6d |
| Wernike et al. (2008) | 125 | DC+DS 30%strain 25° 0.5Hz (5%O2) d2 | bovine | chondrocyte | cylindrical | 6d |
| Wernike et al. (2008) | 125 | DC+DS 30%strain 25° 0.5Hz (21%O2) w2 | bovine | chondrocyte | cylindrical | 6d |
| Wernike et al. (2008) | 125 | DC+DS 30%strain 25° 0.5Hz (5%O2) w2 | bovine | chondrocyte | cylindrical | 6d |
| Wernike et al. (2008) | 125 | DC+DS 30%strain 25° 0.5Hz (21%O2) w4 | bovine | chondrocyte | cylindrical | 6d |
| Wernike et al. (2008) | 125 | DC+DS 30%strain 25° 0.5Hz (5%O2) w4 | bovine | chondrocyte | cylindrical | 6d |
| Wuest et al. (2018) | 126 | CF 10°/s^2 d8 | bovine | chondrocyte | n/a | n/a |
| Wuest et al. (2018) | 126 | CF 10°/s^2 d6 | bovine | chondrocyte | n/a | 2d |
| Xie et al. (2021) | 127 | DC 5%strain 0.2Hz w2 | human | chondrocyte | cylindrical | 14d |
| Xu et al. (2000) | 128 | DT 6%strain 0.05Hz d1 | leporine | chondrocyte | n/a | 6-8d |
| Xu et al. (2000) | 128 | DT 6%strain 0.05Hz (IL1b) d1 | leporine | chondrocyte | n/a | 6-8d |
| Xu et al. (2000) | 128 | DT 6%strain 0.05Hz d2 | leporine | chondrocyte | n/a | 6-8d |
| Xu et al. (2000) | 128 | DT 6%strain 0.05Hz (IL1b) d2 | leporine | chondrocyte | n/a | 6-8d |
| Xu et al. (2000) | 128 | DT 6%strain 0.05Hz d3 | leporine | chondrocyte | n/a | 6-8d |
| Xu et al. (2000) | 128 | DT 6%strain 0.05Hz (IL1b) d3 | leporine | chondrocyte | n/a | 6-8d |
| Yan et al. (2025) | 129 | FIS 100rpm d2 | murine | chondrocyte | cylindrical | n/a |
| Yao et al. (2022) | 130 | HP 5MPa 0.5Hz d1 | human | CPC | spherical | 7d |
| Yu et al. (2015) | 131 | FIS 3ml/min d1 | bovine | chondrocyte | spherical | n/a |
| Yu et al. (2015) | 131 | FIS 3ml/min w1 | bovine | chondrocyte | spherical | n/a |
| Yu et al. (2015) | 131 | FIS 3ml/min w2 | bovine | chondrocyte | spherical | n/a |
| Zhang et al. (2024) | 132 | DT 15° 1Hz (1hr/d) w3 | human | MSC | linear aggregate | 7d |
| Zhang et al. (2024) | 132 | DT 15° 1Hz (6hr/d) w3 | human | MSC | linear aggregate | 7d |
| Zhao et al. (2022) | 133 | DT 10%strain 0.25Hz d3 | human | chondrocyte | n/a | n/a |
| Zhao et al. (2022) | 133 | DT 15%strain 0.25Hz d3 | human | chondrocyte | n/a | n/a |
| Zhao et al. (2022) | 133 | DT 5%strain 0.25Hz d3 | human | chondrocyte | n/a | n/a |
| Zhu et al. (2019) | 134 | DT 25%strain 2Hz w1 | murine | chondrocyte | n/a | 3d |
| Zhu et al. (2019) | 134 | DT 10%strain 1Hz w1 | murine | chondrocyte | n/a | 3d |
| Zhu et al. (2019) | 134 | DT 5%strain 0.5Hz w1 | murine | chondrocyte | n/a | 3d |
| Abbreviation:  DC, direct compression; DS, direct shear; DT, direct tension; HP, hydrostatic pressure; FIS: fluid-induced shear; OP, osmotic pressure; CF, centrifugal force; USVB, ultrasonic vibration | | | | | | |

## Meta-analysis statistics

Table 7. Meta-analysis statistics of effect measures for each mechanical stimulation modality.

| Subgroup | k^a^ | SMD | CI_lb | CI_ub | p value | Tau^2^ | I^2^ |
| --- | --- | --- | --- | --- | --- | --- | --- |
| **Aggrecan Gene Expression** |  |  |  |  |  |  |  |
| Compression | 18 | 0.42 | 0.02 | 0.82 | 4.09E-02 | 0.37 | 52.90 |
| Tension | 38 | 0.56 | -0.27 | 1.39 | 1.83E-01 | 5.88 | 91.02 |
| Shear | 0 | n/a | n/a | n/a | n/a | n/a | n/a |
| Hydrostatic Pressure | 26 | 1.19 | 0.71 | 1.67 | 1.06E-06 | 0.96 | 66.86 |
| Fluid-induced Shear | 13 | -0.56 | -2.00 | 0.89 | 4.49E-01 | 6.11 | 89.46 |
| Compression and Shear | 24 | 2.10 | 1.46 | 2.74 | 1.53E-10 | 1.85 | 78.57 |
| Other Combined Regimes | 17 | -0.66 | -1.99 | 0.66 | 3.27E-01 | 7.10 | 94.64 |
| **Glycosaminoglycan Deposition** |  |  |  |  |  |  |  |
| Compression | 61 | 0.85 | 0.62 | 1.08 | 2.87E-13 | 0.51 | 67.14 |
| Tension | 55 | 0.61 | -0.10 | 1.31 | 9.11E-02 | 6.27 | 92.23 |
| Shear | 8 | 0.60 | 0.17 | 1.04 | 6.40E-03 | 0.15 | 39.00 |
| Hydrostatic Pressure | 38 | 1.33 | 0.90 | 1.77 | 1.57E-09 | 1.23 | 73.62 |
| Fluid-induced Shear | 51 | 0.04 | -0.36 | 0.44 | 8.41E-01 | 1.56 | 77.94 |
| Compression and Shear | 48 | 1.92 | 1.45 | 2.38 | 7.63E-16 | 2.09 | 88.27 |
| Other Combined Regimes | 20 | -0.19 | -0.96 | 0.58 | 6.31E-01 | 2.52 | 87.01 |
| **Collagen II Gene Expression** |  |  |  |  |  |  |  |
| Compression | 15 | -0.02 | -0.78 | 0.74 | 9.53E-01 | 1.82 | 83.67 |
| Tension | 41 | 0.10 | -0.30 | 0.51 | 6.27E-01 | 1.17 | 71.49 |
| Shear | 0 | n/a | n/a | n/a | n/a | n/a | n/a |
| Hydrostatic Pressure | 26 | 0.66 | 0.28 | 1.05 | 7.31E-04 | 0.51 | 55.18 |
| Fluid-induced Shear | 13 | -0.24 | -2.12 | 1.64 | 8.03E-01 | 10.72 | 94.17 |
| Compression and Shear | 32 | 2.33 | 1.55 | 3.11 | 4.75E-09 | 4.15 | 90.03 |
| Other Combined Regimes | 13 | 0.18 | -0.95 | 1.32 | 7.51E-01 | 3.65 | 92.71 |
| **Collagen Deposition** |  |  |  |  |  |  |  |
| Compression | 34 | 0.52 | 0.23 | 0.81 | 3.85E-04 | 0.39 | 60.63 |
| Tension | 5 | 0.41 | -0.84 | 1.67 | 5.20E-01 | 1.42 | 70.42 |
| Shear | 7 | 0.40 | -0.05 | 0.84 | 8.03E-02 | 0.07 | 19.93 |
| Hydrostatic Pressure | 10 | 1.18 | 0.14 | 2.22 | 2.63E-02 | 1.99 | 75.29 |
| Fluid-induced Shear | 35 | 0.29 | -0.10 | 0.69 | 1.47E-01 | 0.97 | 70.75 |
| Compression and Shear | 14 | 2.57 | 1.58 | 3.57 | 3.88E-07 | 2.48 | 88.45 |
| Other Combined Regimes | 4 | -0.12 | -0.66 | 0.43 | 6.80E-01 | n/a | n/a |
| **Compressive Equilibrium Modulus** |  |  |  |  |  |  |  |
| Compression | 11 | 1.33 | 0.34 | 2.33 | 8.46E-03 | 2.34 | 86.70 |
| Tension | 0 | n/a | n/a | n/a | n/a | n/a | n/a |
| Shear | 2 | 1.04 | 0.36 | 1.72 | 2.74E-03 | n/a | n/a |
| Hydrostatic Pressure | 0 | n/a | n/a | n/a | n/a | n/a | n/a |
| Fluid-induced Shear | 17 | 1.51 | 0.75 | 2.27 | 1.02E-04 | 2.02 | 81.77 |
| Compression and Shear | 0 | n/a | n/a | n/a | n/a | n/a | n/a |
| Other Combined Regimes | 3 | 0.77 | -1.12 | 2.66 | 4.26E-01 | 2.39 | 85.83 |
| ^a^ k represents the number of distinct mechanical stimulation groups or protocols. | | | | | | | |

## Risk of bias assessment details


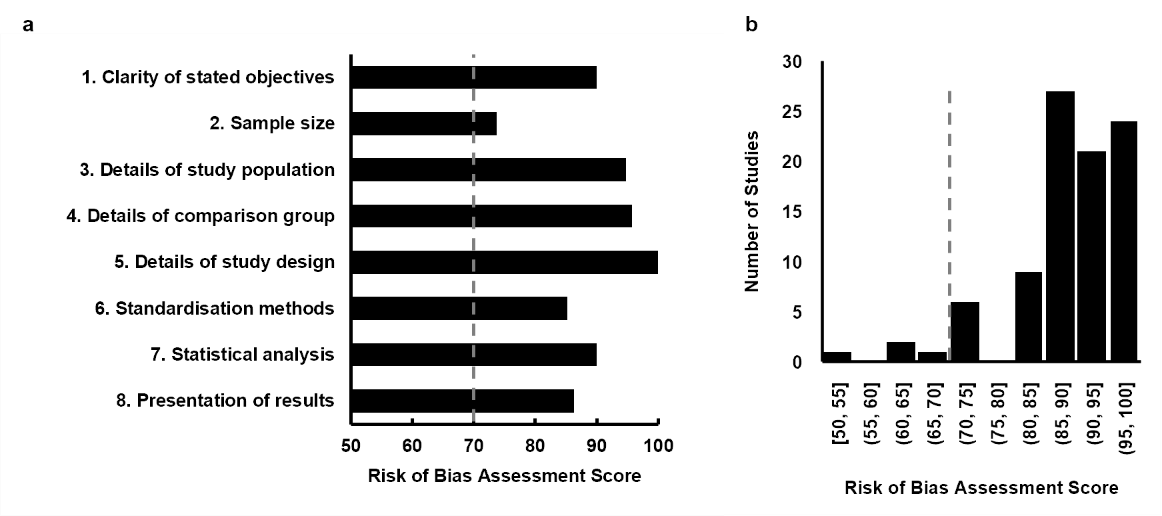


Figure 1. Risk of bias assessment scores across all included studies. (a) Mean scores for each assessment criterion. (b) Distribution of overall risk of bias scores. Dashed line indicates the threshold between low risk (>70%) and medium risk (50-70%) according to the QUIN scale.

## Meta-regression analysis

Table 8. Meta-regression statistics of aggrecan gene expression affected by amplitude.

| Subgroup | Estimate (β) | SE | zval | pval | CI_lb | CI_ub | Tau^2^ | I^2^ |
| --- | --- | --- | --- | --- | --- | --- | --- | --- |
| Compression | -0.04 | 0.02 | -1.55 | 1.22E-01 | -0.08 | 0.01 | 0.28 | 45.21 |
| Tension | -0.10 | 0.08 | -1.27 | 2.05E-01 | -0.26 | 0.06 | 5.89 | 90.91 |
| Hydrostatic Pressure | 0.12 | 0.06 | 2.00 | **4.59E-02** | 0.00 | 0.25 | 0.92 | 65.39 |
| Fluid-induced Shear | 0.65 | 0.31 | 2.11 | **3.46E-02** | 0.05 | 1.25 | 0.25 | 27.19 |


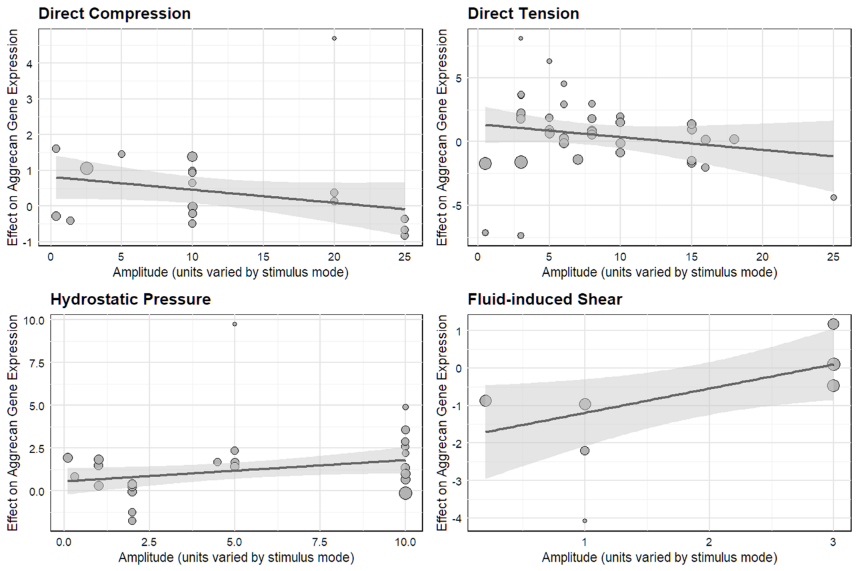


Figure 2. Meta-regression presented in bubble plots, examining the effect of applied amplitude for each stimulation modality on aggrecan gene expression. Stimulus mode with insufficient variations of amplitude was neglected. Each dot represents an individual study effect size with larger dots indicating greater precision (i.e. higher inverse variance). The solid line shows the fitted regression line from the meta-regression model, representing the overall trend of the effect across frequencies, while the shaded ribbon shows the 95% confidence interval.

Table 9. Meta-regression statistics of glycosaminoglycan deposition affected by amplitude.

| Subgroup | Estimate (β) | SE | zval | pval | CI_lb | CI_ub | Tau^2^ | I^2^ |
| --- | --- | --- | --- | --- | --- | --- | --- | --- |
| Compression | 0.00 | 0.01 | 0.18 | 8.60E-01 | -0.02 | 0.02 | 0.39 | 59.75 |
| Tension | -0.08 | 0.04 | -2.05 | **3.99E-02** | -0.15 | 0.00 | 3.65 | 83.06 |
| Shear | -0.10 | 0.05 | -2.04 | **4.09E-02** | -0.20 | 0.00 | 0.00 | 0.00 |
| Hydrostatic Pressure | 0.02 | 0.08 | 0.25 | 8.01E-01 | -0.14 | 0.19 | 1.30 | 66.95 |
| Fluid-induced Shear | -0.03 | 0.11 | -0.26 | 7.97E-01 | -0.24 | 0.18 | 0.72 | 68.15 |


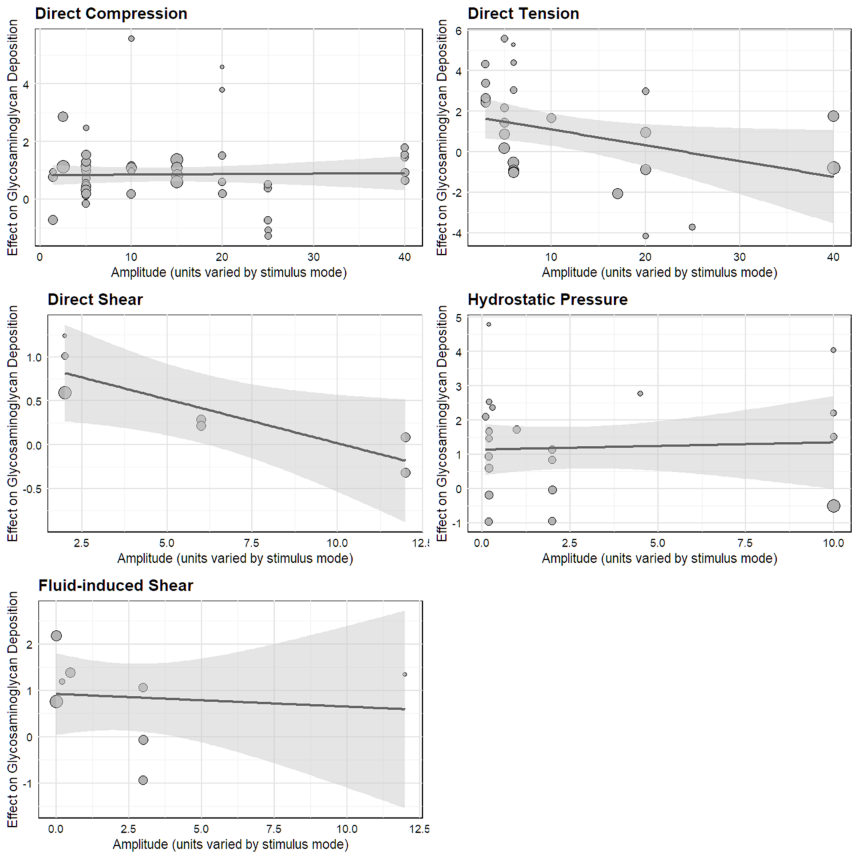


Figure 3. Meta-regression presented in bubble plots, examining the effect of applied amplitude for each stimulation modality on glycosaminoglycan deposition. Each dot represents an individual study effect size with larger dots indicating greater precision (i.e. higher inverse variance). The solid line shows the fitted regression line from the meta-regression model, representing the overall trend of the effect across frequencies, while the shaded ribbon shows the 95% confidence interval.

Table 10. Meta-regression statistics of collagen II gene expression affected by amplitude.

| Subgroup | Estimate (β) | SE | zval | pval | CI_lb | CI_ub | Tau^2^ | I^2^ |
| --- | --- | --- | --- | --- | --- | --- | --- | --- |
| Compression | -0.06 | 0.05 | -1.17 | 2.41E-01 | -0.16 | 0.04 | 1.78 | 82.38 |
| Tension | 0.00 | 0.04 | -0.03 | 9.77E-01 | -0.08 | 0.08 | 1.23 | 72.22 |
| Hydrostatic Pressure | 0.10 | 0.04 | 2.26 | **2.38E-02** | 0.01 | 0.19 | 0.30 | 42.53 |
| Fluid-induced Shear | 0.80 | 1.19 | 0.67 | 5.03E-01 | -1.54 | 3.14 | 8.83 | 87.99 |


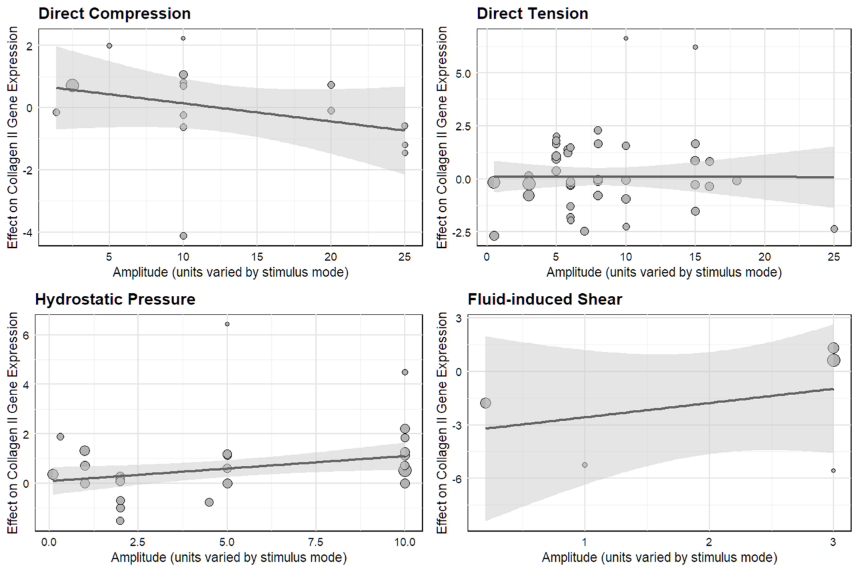


Figure 4. Meta-regression presented in bubble plots, examining the effect of applied amplitude for each stimulation modality on collagen II gene expression. Stimulus mode with insufficient variations of amplitude was neglected. Each dot represents an individual study effect size with larger dots indicating greater precision (i.e. higher inverse variance). The solid line shows the fitted regression line from the meta-regression model, representing the overall trend of the effect across frequencies, while the shaded ribbon shows the 95% confidence interval.

Table 11. Meta-regression statistics of collagen deposition affected by amplitude.

| Subgroup | Estimate (β) | SE | zval | pval | CI_lb | CI_ub | Tau^2^ | I^2^ |
| --- | --- | --- | --- | --- | --- | --- | --- | --- |
| Compression | -0.03 | 0.02 | -1.83 | 6.72E-02 | -0.07 | 0.00 | 0.18 | 43.56 |
| Shear | -0.13 | 0.05 | -2.55 | **1.07E-02** | -0.22 | -0.03 | 0.00 | 0.00 |
| Hydrostatic Pressure | 0.09 | 0.14 | 0.62 | 5.33E-01 | -0.19 | 0.37 | 2.28 | 77.56 |
| Fluid-induced Shear | 0.85 | 0.50 | 1.69 | 9.10E-02 | -0.14 | 1.83 | 2.22 | 84.30 |


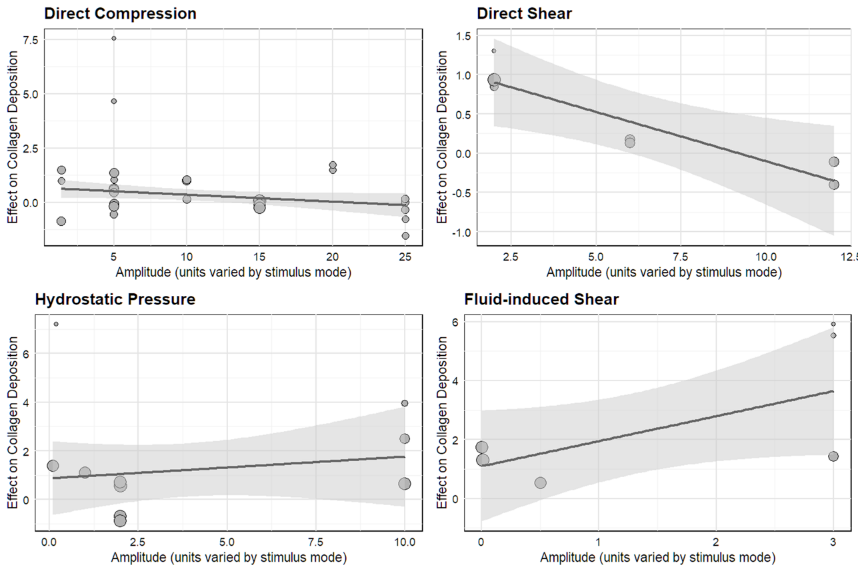


Figure 5. Meta-regression presented in bubble plots, examining the effect of applied amplitude for each stimulation modality on collagen deposition. Stimulus mode with insufficient variations of amplitude was neglected. Each dot represents an individual study effect size with larger dots indicating greater precision (i.e. higher inverse variance). The solid line shows the fitted regression line from the meta-regression model, representing the overall trend of the effect across frequencies, while the shaded ribbon shows the 95% confidence interval.

Table 12. Meta-regression statistics of compressive equilibrium modulus affected by amplitude.

| Subgroup | Estimate (β) | SE | zval | pval | CI_lb | CI_ub | Tau^2^ | I^2^ |
| --- | --- | --- | --- | --- | --- | --- | --- | --- |
| Compression | -0.01 | 0.10 | -0.08 | 9.34E-01 | -0.20 | 0.18 | 2.76 | 88.49 |
| Fluid-induced Shear | -0.36 | 1.33 | -0.27 | 7.88E-01 | -2.97 | 2.25 | 6.93 | 91.50 |


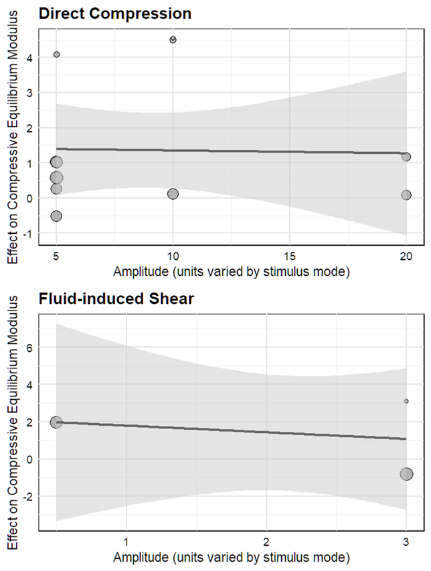


Figure 6. Meta-regression presented in bubble plots, examining the effect of applied amplitude for each stimulation modality on compressive equilibrium modulus. Stimulus mode with insufficient variations of amplitude was neglected. Each dot represents an individual study effect size with larger dots indicating greater precision (i.e. higher inverse variance). The solid line shows the fitted regression line from the meta-regression model, representing the overall trend of the effect across frequencies, while the shaded ribbon shows the 95% confidence interval.

Table 13. Meta-regression statistics of aggrecan gene expression affected by frequency.

| Subgroup | Estimate (β) | SE | zval | pval | CI_lb | CI_ub | Tau^2^ | I^2^ |
| --- | --- | --- | --- | --- | --- | --- | --- | --- |
| Compression | -0.34 | 0.71 | -0.48 | 6.30E-01 | -1.73 | 1.05 | 0.42 | 56.06 |
| Tension | -2.48 | 1.06 | -2.33 | **1.97E-02** | -4.56 | -0.40 | 5.21 | 89.96 |
| Hydrostatic Pressure | -1.62 | 1.56 | -1.04 | 3.00E-01 | -4.68 | 1.45 | 1.00 | 67.94 |
| Fluid-induced Shear | -2.70 | 0.97 | -2.78 | **5.37E-03** | -4.61 | -0.80 | 6.74 | 85.35 |
| Compression and Shear | 4.67 | 0.97 | 4.81 | **1.54E-06** | 2.77 | 6.58 | 0.59 | 53.48 |
| Other Combined Regimes | 2.26 | 3.24 | 0.70 | 4.85E-01 | -4.10 | 8.62 | 7.52 | 94.68 |


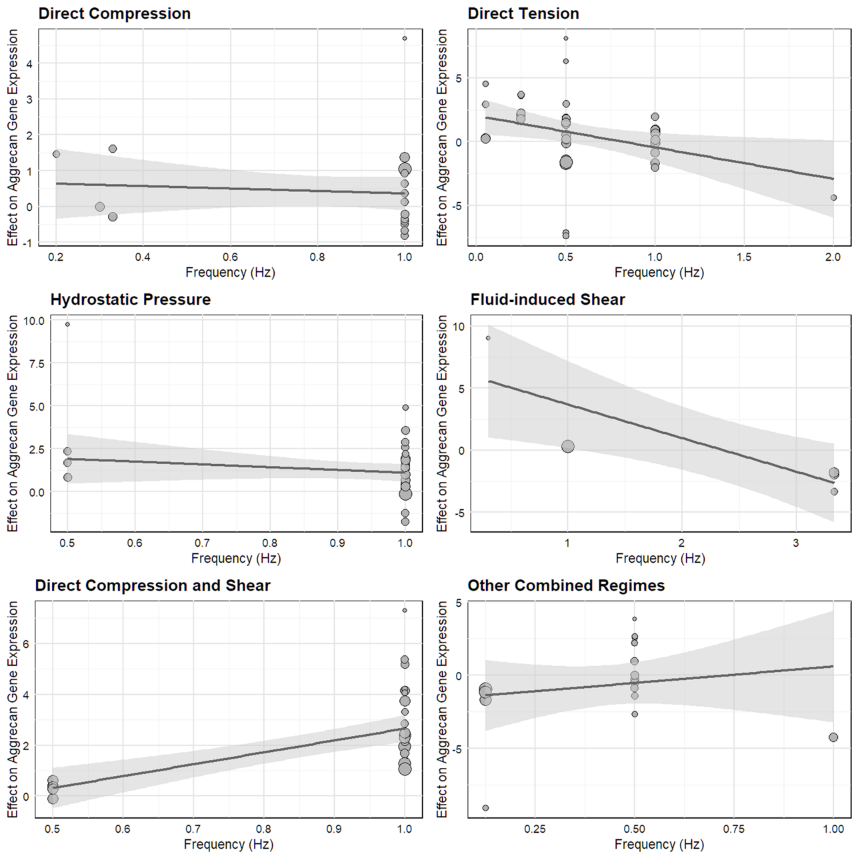


Figure 7. Meta-regression presented in bubble plots, examining the effect of applied frequency (Hz) of fluid-induced shear on aggrecan gene expression. Stimulus mode with insufficient variations of frequency was neglected. Each dot represents an individual study effect size with larger dots indicating greater precision. The solid line shows the fitted regression line from the meta-regression model, representing the overall trend of the effect across frequencies, while the shaded ribbon shows the 95% confidence interval.

Table 14. Meta-regression statistics of glycosaminoglycan deposition affected by frequency.

| Subgroup | Estimate (β) | SE | zval | pval | CI_lb | CI_ub | Tau^2^ | I^2^ |
| --- | --- | --- | --- | --- | --- | --- | --- | --- |
| Compression | -0.04 | 0.31 | -0.13 | 8.97E-01 | -0.65 | 0.57 | 0.38 | 58.17 |
| Tension | -0.62 | 0.88 | -0.71 | 4.80E-01 | -2.35 | 1.10 | 3.93 | 84.48 |
| Hydrostatic Pressure | -0.12 | 0.65 | -0.19 | 8.50E-01 | -1.39 | 1.15 | 1.28 | 68.08 |
| Fluid-induced Shear | -0.41 | 0.28 | -1.51 | 1.32E-01 | -0.95 | 0.12 | 0.67 | 62.40 |
| Compression and Shear | -3.96 | 1.98 | -2.00 | **4.59E-02** | -7.84 | -0.07 | 2.17 | 88.17 |
| Other Combined Regimes | 2.84 | 1.44 | 1.97 | **4.88E-02** | 0.01 | 5.66 | 0.41 | 52.70 |


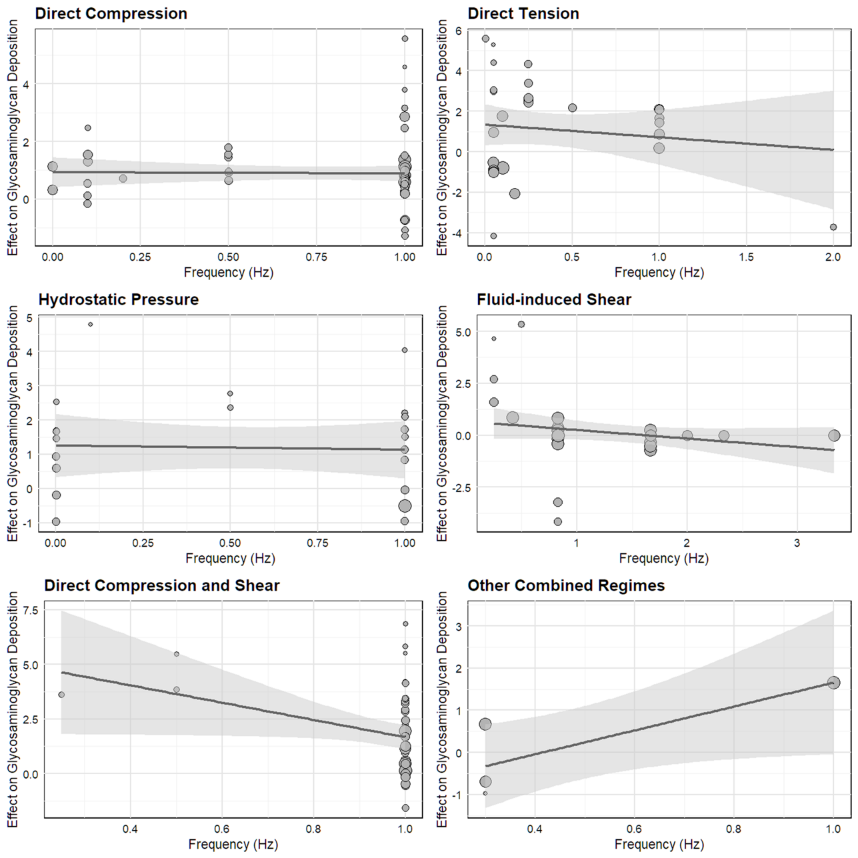


Figure 8. Meta-regression presented in bubble plots, examining the effect of applied frequency (Hz) of fluid-induced shear on glycosaminoglycan deposition. Stimulus mode with insufficient variations of frequency was neglected. Each dot represents an individual study effect size with larger dots indicating greater precision. The solid line shows the fitted regression line from the meta-regression model, representing the overall trend of the effect across frequencies, while the shaded ribbon shows the 95% confidence interval.

Table 15. Meta-regression statistics of collagen II gene expression affected by frequency.

| Subgroup | Estimate (β) | SE | zval | pval | CI_lb | CI_ub | Tau^2^ | I^2^ |
| --- | --- | --- | --- | --- | --- | --- | --- | --- |
| Compression | -2.36 | 1.46 | -1.62 | 1.06E-01 | -5.22 | 0.50 | 1.60 | 81.58 |
| Tension | 0.31 | 0.54 | 0.58 | 5.59E-01 | -0.74 | 1.37 | 1.17 | 71.50 |
| Hydrostatic Pressure | -2.48 | 1.21 | -2.05 | **4.01E-02** | -4.86 | -0.11 | 0.68 | 62.33 |
| Fluid-induced Shear | -1.92 | 0.82 | -2.35 | **1.89E-02** | -3.53 | -0.32 | 8.16 | 93.08 |
| Compression and Shear | 6.01 | 1.55 | 3.87 | **1.10E-04** | 2.96 | 9.05 | 2.42 | 83.93 |
| Other Combined Regimes | -0.49 | 2.47 | -0.20 | 8.42E-01 | -5.34 | 4.35 | 4.21 | 93.52 |


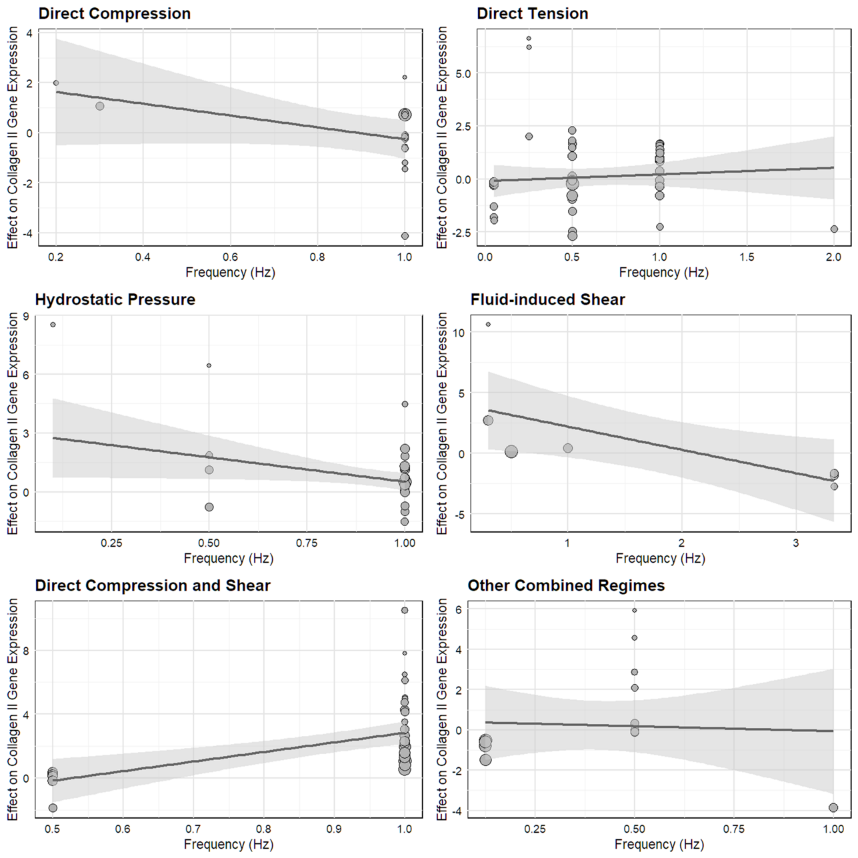


Figure 9. Meta-regression presented in bubble plots, examining the effect of applied frequency (Hz) of fluid-induced shear on collagen II gene expression. Stimulus mode with insufficient variations of frequency was neglected. Each dot represents an individual study effect size with larger dots indicating greater precision (i.e. higher inverse variance). The solid line shows the fitted regression line from the meta-regression model, representing the overall trend of the effect across frequencies, while the shaded ribbon shows the 95% confidence interval.

Table 16. Meta-regression statistics of collagen protein deposition affected by frequency.

| Subgroup | Estimate (β) | SE | zval | pval | CI_lb | CI_ub | Tau^2^ | I^2^ |
| --- | --- | --- | --- | --- | --- | --- | --- | --- |
| Compression | -0.22 | 0.40 | -0.55 | 5.79E-01 | -1.00 | 0.56 | 0.43 | 62.48 |
| Tension | -2.52 | 0.87 | -2.91 | **3.59E-03** | -4.22 | -0.82 | 0.01 | 1.46 |
| Hydrostatic Pressure | -7.16 | 2.33 | -3.07 | **2.14E-03** | -11.72 | -2.59 | 0.69 | 53.61 |
| Fluid-induced Shear | -0.57 | 0.28 | -2.00 | **4.58E-02** | -1.12 | -0.01 | 0.46 | 54.27 |
| Compression and Shear | -0.03 | 1.82 | -0.02 | 9.87E-01 | -3.60 | 3.54 | 2.87 | 90.16 |
| Other Combined Regimes | 0.72 | 0.86 | 0.83 | 4.05E-01 | -0.97 | 2.40 | 0.00 | 0.00 |


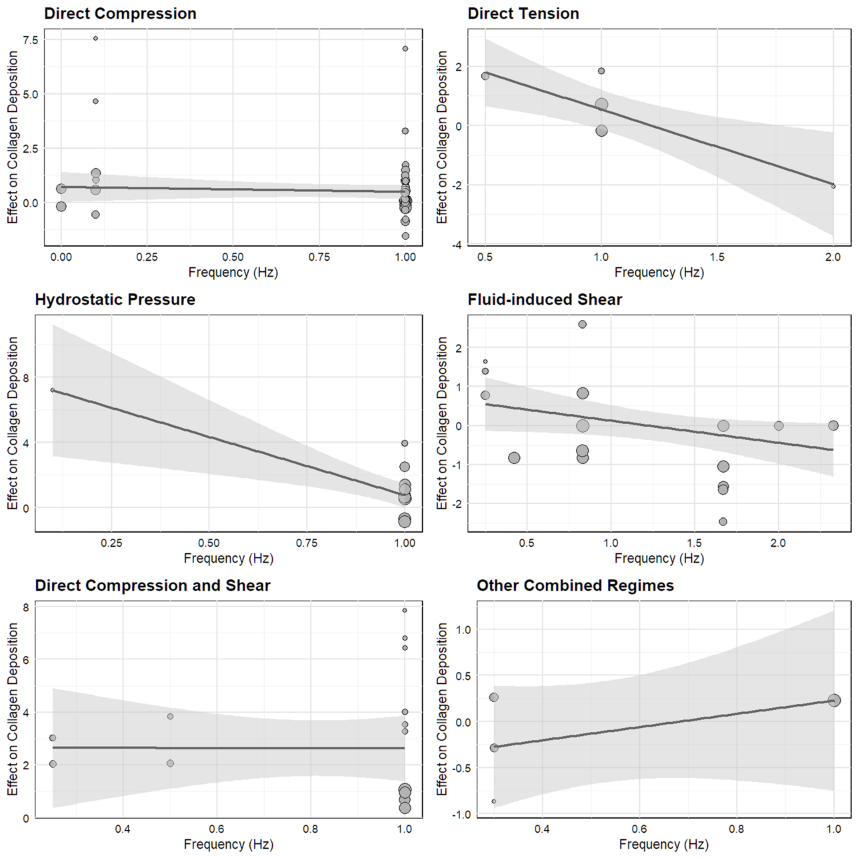


Figure 10. Meta-regression presented in bubble plots, examining the effect of applied frequency (Hz) of fluid-induced shear on collagen protein deposition. Stimulus mode with insufficient variations of frequency was neglected. Each dot represents an individual study effect size with larger dots indicating greater precision (i.e. higher inverse variance). The solid line shows the fitted regression line from the meta-regression model, representing the overall trend of the effect across frequencies, while the shaded ribbon shows the 95% confidence interval.

Table 17. Meta-regression statistics of compressive equilibrium modulus affected by frequency.

| Subgroup | Estimate (β) | SE | zval | pval | CI_lb | CI_ub | Tau^2^ | I^2^ |
| --- | --- | --- | --- | --- | --- | --- | --- | --- |
| Fluid-induced Shear | -2.00 | 0.83 | -2.43 | **1.52E-02** | -3.62 | -0.39 | 1.21 | 71.78 |
| Other Combined Regimes | 4.02 | 1.36 | 2.96 | **3.08E-03** | 1.36 | 6.68 | 0.14 | 28.93 |


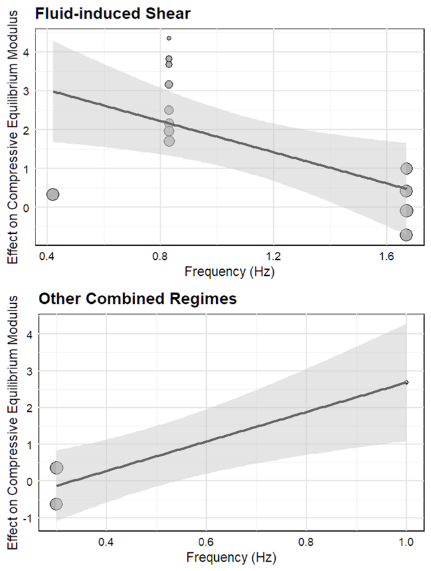


Figure 11. Meta-regression presented in bubble plots, examining the effect of applied frequency (Hz) of fluid-induced shear on compressive equilibrium modulus. Stimulus mode with insufficient variations of frequency was neglected. Each dot represents an individual study effect size with larger dots indicating greater precision (i.e. higher inverse variance). The solid line shows the fitted regression line from the meta-regression model, representing the overall trend of the effect across frequencies, while the shaded ribbon shows the 95% confidence interval.

## Recommended Reporting Checklist

Table 18. Recommended reporting for *in vitro* cartilage tissue engineering

| Category | Reporting Item | Recommended Documentation Requirement |
| --- | --- | --- |
| Cell Source | Cell demographics | Report species of cell source, donor age, biological sex, and anatomical harvest site. |
|  | Cell handling | Report passage number at the time of seeding, seeding density, and pre-culture condition. |
| Tissue Construct | Scaffold design | Specify whether the tissue model is scaffold-free or not, scaffold material, crosslinking method, and macroscopic dimension (mm). |
|  | Microarchitecture | Report quantifiable morphological parameter such as porosity (%), average pore size (µm), and strut topology. |
| Culture Condition | Sample size | Specify the number of biological replicates (e.g., distinct human donors or animal batches) and technical replicates (e.g., number of engineered constructs per donor) per experimental group. |
|  | Culture environment | Report temperature (°C), relative humidity (%), O₂ concentration (%), and CO₂ concentration (%). |
|  | Culture medium | Report the base medium formulation (e.g., DMEM high glucose) and specify whether serum (include percentage and lot number) or serum-free conditions were used. |
|  | Supplement usage | Report all added supplements, such as growth factors, dexamethasone, ascorbic acid, ITS. |
| Bioreactor Specification | System architecture | Specify whether the system is commercial or custom-built, details of the actuation mechanism such as stepper motor, peristaltic pump, etc. |
|  | Component variability | Report accuracy and resolution of electronics, and engineering tolerances for mechanical parts. |
|  | Functional verification | Specify the functional verification or calibration performed such as actuator performance verification, sensor sensitivity, assembly tolerance stack-up analysis. |
| Mechanical Stimulation | Loading modality | Define the primary modality of the stimulation used in the group such as compression, tension, shear, hydrostatic pressure, fluid-induced shear. |
|  | Static parameter | Report static pre-load (N), pre-strain (%), or static pressure offset (Pa). |
|  | Dynamic parameter | Report the peak dynamic amplitude with a corresponding unit (e.g., % strain for compression, tension, and shear), operating frequency (Hz), strain rate (%/s), and loading profile in terms of the waveform shape (e.g., sinusoidal, square wave). |
|  | Stimulation duration | Specify the daily duty cycle (e.g., 1 hour on / 23 hour off), total culture duration (days). |
| Chondrogenic Assessment | Gene expression | Quantify mRNA expression of positive transcriptional biomarkers (e.g., *ACAN*, *COL2A1*, *SOX9*) and negative/hypertrophic biomarkers (e.g., *COL1A1*, *COL10A1*, *RUNX2*). |
|  | Matrix deposition | Quantify total accumulated matrix with biochemical assays (e.g., sGAG/DNA). |
|  | Normalisation | Specify the housekeeping genes for qPCR analysis, the comparative method, normalisation references for biochemical assays. |
|  | Mechanical property | Evaluate the macroscopic mechanical property of the engineered construct (e.g., compressive equilibrium modulus), and report parameters used for the mechanical test (e.g., strain rate, pre-load, stepwise load, step number, calculation method for modulus, etc.) |
